# Heme pathway evolution in kinetoplastid protists

**DOI:** 10.1186/s12862-016-0664-6

**Published:** 2016-05-18

**Authors:** Ugo Cenci, Daniel Moog, Bruce A. Curtis, Goro Tanifuji, Laura Eme, Julius Lukeš, John M. Archibald

**Affiliations:** Department of Biochemistry and Molecular Biology, Dalhousie University, Halifax, Canada; Centre for Comparative Genomics and Evolutionary Bioinformatics, Halifax, Nova Scotia Canada; Faculty of Life and Environmental Sciences, University of Tsukuba, Tsukuba, Japan; Institute of Parasitology, Biology Centre, Czech Academy of Sciences, and Faculty of Sciences, University of South Bohemia, České Budӗjovice, Czech Republic; Canadian Institute for Advanced Research, Toronto, Canada

**Keywords:** Heme, Kinetoplastea, *Paramoeba pemaquidensis*, *Perkinsela*, Evolution, Endosymbiosis, Prokinetoplastina, Lateral gene transfer

## Abstract

**Background:**

Kinetoplastea is a diverse protist lineage composed of several of the most successful parasites on Earth, organisms whose metabolisms have coevolved with those of the organisms they infect. Parasitic kinetoplastids have emerged from free-living, non-pathogenic ancestors on multiple occasions during the evolutionary history of the group. Interestingly, in both parasitic and free-living kinetoplastids, the heme pathway—a core metabolic pathway in a wide range of organisms—is incomplete or entirely absent. Indeed, Kinetoplastea investigated thus far seem to bypass the need for heme biosynthesis by acquiring heme or intermediate metabolites directly from their environment.

**Results:**

Here we report the existence of a near-complete heme biosynthetic pathway in *Perkinsela* spp., kinetoplastids that live as obligate endosymbionts inside amoebozoans belonging to the genus *Paramoeba*/*Neoparamoeba*. We also use phylogenetic analysis to infer the evolution of the heme pathway in Kinetoplastea.

**Conclusion:**

We show that *Perkinsela* spp. is a deep-branching kinetoplastid lineage, and that lateral gene transfer has played a role in the evolution of heme biosynthesis in *Perkinsela* spp. and other Kinetoplastea. We also discuss the significance of the presence of seven of eight heme pathway genes in the *Perkinsela* genome as it relates to its endosymbiotic relationship with *Paramoeba*.

**Electronic supplementary material:**

The online version of this article (doi:10.1186/s12862-016-0664-6) contains supplementary material, which is available to authorized users.

## Background

Kinetoplastea is a diverse group of unicellular flagellated organisms, most of which are parasites. The best known group of kinetoplastid parasites is the Trypanosomatida, which parasitize plants (e.g., *Phytomonas* [1]), insects (e.g., *Angomonas* [2]) and humans (e.g., *Leishmania* [3] and *Trypanosoma* [4]). However, the Kinetoplastea also includes non-parasitic organisms such as free-living bodonids like *Bodo saltans* [5] and *Neobodo designis* [6]. The bodonids are comprised of Neobodonida, Eubodonida and Parabodonida, which are considered early branching Kinetoplastea [7–10] and serve as an important reference point for the evolution of parasitism within this species-rich group [11]. However, these organisms are poorly understood and the evolutionary relationship amongst bodonids is still debated [8, 10, 12]. The Prokinetoplastina is an even deeper branching group of kinetoplastid flagellates [7–9], and is composed of organisms such as *Ichthyobodo necator,* a fish ectoparasite, and *Perkinsela* sp. [13], which is an endosymbiont of opportunistic pathogenic amoebae belonging to *Neoparamoeba*/*Paramoeba* spp. [14–18]. The Kinetoplastea themselves belong to the Excavata, more specifically the Euglenozoa, which includes Diplonemida (e.g., *Diplonema papillatum*) and Euglenida such as the plastid-bearing organisms *Eutreptiella gymnastica* and *Euglena gracilis*.

Parasitic Kinetoplastea have complex life cycles and have undergone extensive reductive evolution as a consequence of their parasite-host interactions. One commonly observed change is the reduction or complete loss of biochemical pathways that can be augmented or provided by their hosts [11]. This includes the lack of tetrahydrobiopterin biosynthesis required for folate and pteridine [19, 20] and, in trypanosomatids, purine auxotrophy [21]. One particularly striking example of such metabolic reduction in Kinetoplastea is the heme pathway, which is either incomplete or missing entirely (in some parasitic species). In the latter case, essential metabolites are acquired from their hosts [22, 23] or, in the case of *Strigomonas culicis* and *Angomonas deanei*, from bacterial endosymbionts [24]. Metabolite import could involve intermediates in the heme pathway from coproporphyrinogen III, as suggested by Kořenỳ et al. [25], or heme itself [26, 27]. Furthermore, the plant pathogen *Phytomonas serpens* seems not to require heme at all [28]. The heme pathway is not found in *Trypanosoma* and only the last three steps of the pathway are present in other trypanosomatids such as Leishmaniinae (composed of *Leptomonas* spp., *Crithidia* spp. and *Leishmania* spp.) [29] and Strigomonadinae (composed of *Angomonas* spp. and *Strigomonas* spp.). No complete heme pathway has been described for a member of the Kinetoplastea [25].

**Fig. 1:**
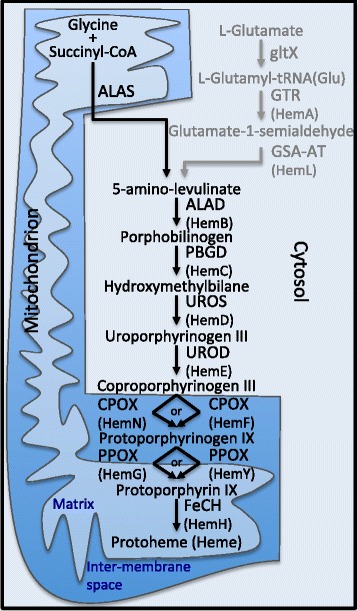
Heme pathway in eukaryotic cells. The Heme pathway in Opisthokonta, and most likely other heterotrophic eukaryotes, as described in Kořenỳ et al. [25], is represented in black. An alternate entry to the pathway, present in bacteria and in the plastids of algae, is represented in grey. For each protein name the corresponding protein in bacteria is indicated in parenthesis. The heme pathway in eukaryotes takes place in the cytosol for the steps involving ALAD, PBGD, UROS, and UROD, while ALAS and FeCH act in the mitochondrial matrix, and the PPOX and CPOX act in the inter-membrane space. Abbreviations: ALAS: 5-aminolevulinate synthase, GltX: glutamyl-tRNA synthetase, GTR: glutamyl-tRNA reductase, GSA-AT: glutamate-1-semialdehyde 2,1-aminomutase, ALAD: delta-aminolevulinic acid dehydratase, PBGD: porphobilinogen deaminase, UROS: uroporphyrinogen-III synthase, UROD: uroporphyrinogen decarboxylase, CPOX/HemF: coproporphyrinogen III oxidase, CPOX/HemN: oxygen-independent coproporphyrinogen III oxidase, PPOX/HemY: oxygen-dependent protoporphyrinogen oxidase, PPOX/HemG: menaquinone-dependent protoporphyrinogen oxidase, FeCH: ferrochelatase

The heme pathway is an important part of cellular metabolism. It produces the cofactor heme, which is required for key biochemical processes such as oxidative phosphorylation. In most eukaryotes, heme biosynthesis involves eight steps (Fig. 1), the first being the transformation of glycine or L-glutamate into 5-amino-levulinate by 5-aminolevulinate synthase (ALAS) [30]. An alternative is the synthesis of 5-amino-levulinate by the sequential action of glutamyl-tRNA synthetase (GltX), glutamyl-tRNA reductase (GTR), and glutamate-1-semialdehyde 2,1-aminomutase (GSA-AT). This second pathway is found in most bacteria and in most eukaryotes with a plastid [31, 32]. 5-amino-levulinate is then converted into porphobilinogen by porphobilinogen synthase (also known as delta-aminolevulinic acid dehydratase (ALAD)), and then into hydroxymethylbilane by hydroxymethylbilane synthase (or porphobilinogen deaminase (PBGD)). Subsequently, uroporphyrinogen-III synthase (UROS) produces uroporphyrinogen III, which is then converted into coproporphyrinogen III by uroporphyrinogen decarboxylase (UROD) [33, 34].

In the next step of the heme pathway, coproporphyrinogen III is modified to protoporphyrinogen IX by coproporphyrinogen III oxidase (CPOX/HemF) under aerobic conditions, and under anaerobic conditions, by oxygen-independent coproporphyrinogen III oxidase (CPOX/HemN) [35]. Subsequently, protoporphyrinogen IX is transformed into protoporphyrin IX by oxygen-dependent protoporphyrinogen oxidase (PPOX/HemY), or menaquinone-dependent protoporphyrinogen oxidase (PPOX/HemG), the latter enzyme possessing the ability to perform the reaction under aerobic and anaerobic conditions [36–38]. Finally, protoporphyrin IX is converted to protoheme by the action of ferrochelatase (FeCH) [39]. Interestingly, the heme biosynthetic pathway in eukaryotes involves proteins in three different cellular locations. While the ALAD, PBGD, UROS, and UROD enzymes act in the cytosol, the ALAS and FeCH enzymes are usually localized to the mitochondrial matrix, and PPOX and CPOX function in the inter-membrane space of the mitochondrion [25, 40] (Fig. 1).

In this study, we analyzed the heme pathway of two species of *Perkinsela*, which are endosymbionts of the amoebozoans *Paramoeba pemaquidensis* and *P. invadens*. Using molecular phylogenetics, we show that *Perkinsela* belongs to the earliest branching kinetoplastid lineage currently known, and demonstrate that these highly reduced endosymbionts nevertheless possess a near-complete heme biosynthesis pathway, the first of its kind described for a member of the Kinetoplastea. We hypothesize that at least a subset of these enzymes represents elements of the ancestral heme pathway in the group. Finally, we discuss the importance of this pathway in early-branching kinetoplastid flagellates for understanding the adaptations that have occurred during the evolution of bodonids and parasitic trypanosomatids.

## Methods

### Heme pathway protein sequence acquisition

We searched for genes encoding heme biosynthetic enzymes in the nuclear genome of the *Perkinsela* endosymbiont living within the amoebozoan *Paramoeba pemaquidensis* CCAP1560/4. The nuclear genome (and associated transcriptome) of the host *P. pemaquidensis* was also queried, as was transcriptomic data from another species, *P. invadens*. GenBank accession numbers for the *Perkinsela* spp. and *Paramoeba* spp. data used in this study are LFNC00000000 and KU609011-KU609043. BLASTp/tBLASTn searches were carried out using a curated set of heme pathway enzymes from diverse eukaryotes as queries with an E-value cut-off 1e-05. For identification of UROS enzymes, local HMMER [41] searches (*hmmsearch*) were initially performed against the total *P. invadens* transcriptome database (6-frame translation into protein) using default settings. Profile HMMs were constructed via *hmmbuild* with Stockholm alignments (‘Seed’ and ‘NCBI’) for HEM4 (PF02602) retrieved from the Pfam website (http://pfam.xfam.org). Hits with an E-value ≤ 1e-05 were then used as queries to screen the *P. pemaquidensis* total genomic and transcriptomic assemblies via local tBLASTn. Homologs were also identified using Ghostkoala (http://www.kegg.jp/ghostkoala/). Sequences used in phylogenetic analyses were obtained by BLAST from the NCBI nr database, the MMETSP database of transcriptomes [42], from TritrypDB [43], and from the *B. saltans* genome (Welcome Trust Sanger Institute). To further verify their predicted roles in heme biosynthesis, all sequences analyzed in this study were annotated using InterProScan [44] and the InterPro classification [45].

### Protein localization predictions

Sequences of proteins putatively involved in heme biosynthesis were subjected to a localization prediction pipeline using the following tools: SignalP 3.0 (http://www.cbs.dtu.dk/services/SignalP-3.0/) [46], TargetP 1.1 (http://www.cbs.dtu.dk/services/TargetP/) [47], PredSL (http://aias.biol.uoa.gr/PredSL/input.html) [48] and Predotar (https://urgi.versailles.inra.fr/predotar/predotar.html) [49] with standard settings for prediction of N-terminal targeting signals such as secretory signal peptides (SPs) and mitochondrial targeting peptides (mTPs). TMHMM 2.0 (http://www.cbs.dtu.dk/services/TMHMM/) [50] was used for analysis of potential transmembrane domains (TMDs). Euglenophytes harbor complex three membrane-bound plastids of green algal origin and the proteins targeted to these organelles usually possess bipartite targeting signals (BTS) consisting of a SP followed by a transit peptide (TP) [51, 52]. Plastid targeting of proteins in photosynthetic euglenids was thus predicted using TargetP, PredSL and Predotar (see above) in ‘plant/chloroplast’ mode after removal of the signal peptide (SP) (based on SignalP prediction). For classification of a protein into one of four categories (secretory, mitochondrial, plastidial, ‘other’), the output of at least two of the searches had to be positive for a specific category (see Additional file 1: Table S1). Only those protein sequences starting with a methionine residue were classified.

### Phylogenetic analysis

Eleven proteins were selected for their potential to resolve the evolution of Kinetoplastea in general and the taxonomic position of *Perkinsela* spp. in particular (Additional file 2: Table S2; proteins used for *Perkinsela* sp. from *P. pemaquidensis* were: KNH09580, KNH04116, KNH06333, KNH09360, KNH08922, KNH06227, KNH03620, KNH06559, KNH08032, KNH06818, KNH05478). Phylogenetic trees were first constructed individually for each protein. Homologs were aligned using MUSCLE [53] and blocks were selected using BMGE [54] with the BLOSUM40 similarity matrix and a block size of four. Each individual protein tree was obtained with IQ-TREE using the ultrafast bootstrap method under the LG4X model and was checked manually before concatenation. Sequences for each organism were then concatenated (30 taxa, 5,060 sites), and a single phylogenetic tree was built using Phylobayes version 4.1 [55] under the catfix C20 + Poisson model [56]. The two chains were stopped when convergence was reached (maxdiff < 0.1) after 230 cycles and a burn-in of 300 trees. We then mapped bootstrap values obtained from 1,000 replicates under the LG4X [57] model with IQ-TREE software [58]. Trees were visualized using Figtree (http://tree.bio.ed.ac.uk/software/figtree/). Topological tree tests were performed using RAxML version 8.0.19 [59] under the PROTGAMMALG4X model. The different topologies were then compared according to the tree topology test available in IQ-TREE (RELL approximation [60], the Kishino-Hasegawa test [61], the Shimodaira-Hasegawa test [62], and expected likelihood weights [63]). The percentage of missing data for each organism in the concatenated alignment is provided in Additional file 3: Table S1.4.

We also built phylogenetic trees for enzymes involved in the heme pathway in Kinetoplastea. Sequences were retrieved using homology searches by BLAST against sequences obtained from different sources (see above). All sequences with an E-value less than 1e-5 were selected. We then aligned these sequences using MAFFT with the fast alignment settings [64]. Block selection was then performed using BMGE with a block size of 4 and the BLOSUM30 similarity matrix. Preliminary trees were generated using Fasttree [65] and ‘dereplication’ was applied to robustly supported monophyletic clades using TreeTrimmer [66] in order to reduce sequence redundancy. For each protein, the final set of sequences was selected manually. Proteins were re-aligned with MUSCLE, block selection was carried out using BMGE with a block size of four and the matrix BLOSUM30, and trees were generated using Phylobayes-4.1 under the catfix C20 + Poisson model with the two chains stopped when convergence was reached (maxdiff < 0.1) after at least 200 cycles, discarding 1,000 burn-in trees. Bootstrap support values were estimated from 100 replicates using IQ-TREE under the LG4X model and mapped onto the Bayesian tree.

## Results

### *Perkinsela*: an early branching kinetoplastid lineage

**Fig. 2:**
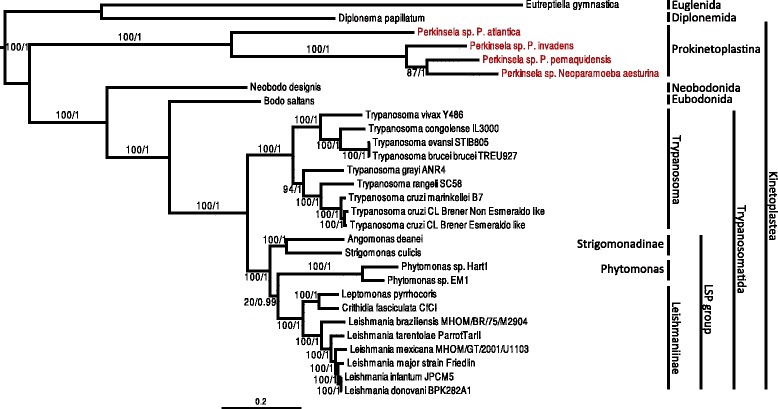
Phylogeny of Kinetoplastea based on a concatenation of 11 proteins. The tree was built using the C20 + Poisson model with Phylobayes 4.1. 1,000 bootstrap replicates were performed using the LG4X model IQTREE and mapped onto the nodes as percentages (*left*) alongside Bayesian posterior probabilities (*right*). Bootstrap values >50 % are shown, while only posterior probabilities >0.6 are shown. The topology of this tree, rooted with Diplonemida and Euglenida, is the same as in Additional file 3: Figure S1.1, which includes a more distantly related outgroup, *Naegleria gruberi*. In both trees, Prokinetoplastina (highlighted red) are the earliest branching kinetoplastid lineage. The bodonids branch as sister to the Trypanosomatida, while Leishmaniinae, *Phytomonas* and Strigomonadinae form a strongly supported, distinct group. Higher-level taxonomic classifications are indicated on the right for each group of organisms. The scale bar indicates the inferred number of substitutions per amino acid site

Together with Euglenida and Diplonemida, Kinetoplastea belong to the Euglenozoa. We built a phylogeny of eleven concatenated proteins (Fig. 2 and Additional file 3: Figure S1.1), sampled from the Prokinetoplastina containing the *Perkinsela* spp. group, with representatives from trypanosomatids and bodonids, and rooted with the diplonemid *Diplonema papillatum*, the euglenid *Eutreptiella gymnastica* and the heterolobosean *Naegleria gruberi*. The eleven proteins were carefully selected based on their availability in public databases in the lineages of interest (i.e., *Eutreptiella gymnastica*, *Diplonema papillatum*, *Perkinsela* spp., *Neobodo designis* and *Bodo saltans*) so as to minimize missing data in our supermatrix. Our results confirm that the diversity of *Perkinsela* spp. for which genomic and/or transcriptomic sequence data are currently available represent a monophyletic assemblage, as previously described [16]. We tested the effects of a distantly related outgroup by removing *N. gruberi* to see if the Prokinetoplastina was still positioned as the deepest branch of the Kinetoplastea (Fig. 2). We then ran topology tests to assess the early branching nature of *Perkinsela* spp. within Kinetoplastea, and to determine if alternative topologies to the Trypanosomatida clade formed by *Trypanosoma*, *Phytomonas*, the Strigomonadinae and Leishmaniinae were rejected (Additional file 3: Table S1.2). Our results suggest that the *Perkinsela* spp. group represents a monophyletic deep branching clade, positioned between the diplonemids and bodonids. While there is some uncertainty with the position of Prokinetoplastina relative to Diplonema, the Kishino-Hasegawa test rejected the possibility that the former branches deeper in the tree than the latter (Additional file 3: Table S1.2). Moreover, the group formed by *Phytomonas*, the Strigomonadinae and Leishmaniinae was found to be robust. However, the relative branching of Strigomonadinae, *Phytomonas* and Leishmaniinae is not clear.

### A near-complete set of heme pathway enzymes in *Perkinsela* spp.

Unlike other kinetoplastid flagellates, and despite having a reduced genome due to its endosymbiotic lifestyle (Tanifuji et al., unpublished data), with the exception of one protein (UROS, see below), we found a full set of heme biosynthesis enzymes encoded in the nuclear genome of the *Perkinsela* sp. living within the amoeba *Paramoeba pemaquidensis*. We also analyzed genes for heme pathway enzymes in the transcriptomes of *Paramoeba atlantica, P. invadens*, and *Neoparamoeba aestuarina*. For each of these species, the sequence data are a mixture of host- and endosymbiont-derived transcripts, due to the fact that it is not possible to separate the *Perkinsela* endosymbionts from their amoeba hosts. Despite this complication, our results are consistent with the existence of an almost complete heme pathway in the *Perkinsela* sp. endosymbionts within *P. invadens* and *N. aestuarina*. In the case of the *Perkinsela* sp. of *P. atlantica*, genes for only two enzymes were identified, perhaps due to the limited number of endosymbiont-derived transcripts in the data.

Beyond *Perkinsela* spp., we inferred the presence/absence of heme biosynthesis enzymes in all the organisms present in the phylogenetic tree shown in Fig. 2, as well as for the amoebozoan hosts in which *Perkinsela* spp. reside (Table 1 and Additional file 4: Table S3). Our results are consistent with those of others [24, 25, 28] in showing the complete absence of the heme pathway in members of the genus *Trypanosoma*. The Strigomonadinae/Leishmaniinae group was found to possess only the last three steps of heme biosynthesis, while *Phytomonas* spp. appear to have only the ferrochelatase (FeCH) enzyme. However, we also found a potential uroporphyrinogen-III synthase enzyme in Leishmaniinae, which has not previously been discussed. In addition, we identified an almost complete heme pathway in the euglenid *E. gymnastica*, and, as expected for a plastid-bearing organism, the alga-associated GTR and GSA-AT enzymes [67]. We found no evidence for the existence of a heme biosynthesis pathway in the diplonemid *Diplonema papillatum*, noting that at present only a small amount of sequence data is publicly available.

**Table 1:** Presence/absence of enzymes involved in the biosynthesis of heme

		ALAS	gltX	GTR (HemA)	GSA-AT (HemL)	ALAD (HemB)	PBGD (HemC)	UROS (HemD)	UROD (HemE)	CPOX (HemF)	CPOX (HemN)	PPOX (HemY)	PPOX (HemG)	FeCH (HemH)
Naegleria	*Naegleria gruberi*		+		+						+			+
Euglenida	*Eutreptiella gymnastica*	+	+	+	+	+	+	+	+	+	+	+		+
Diplonemida	*Diplonema papillatum* *													
Prokinetoplastina	putative *Perkinsela* sp. from *Paramoeba atlantica*		+											
	putative *Perkinsela* sp*.* from *Neoparamoeba* aestuarina	+	+			+	+		+	+		+		+
	*Perkinsela* sp*.* from *P. pemaquidensis* CCAP1560	+	+			+	+		+	+		+		+
	*Perkinsela* sp*.* from *P. invadens*	+	+			+	+		+	+		+		+
Neobodonida	*Neobodo designis*		+											
Eubodonida	*Bodo saltans*		+											
Trypanosoma	*Trypanosoma brucei* TREU927		+											
	*Trypanosoma congolense* IL3000		+											
	*Trypanosoma cruzi* CL Brener Esmeraldo-like		+											
	*Trypanosoma cruzi* CL Brener Non-Esmeraldo-like		+											
	*Trypanosoma cruzi marinkellei* B7		+											
	*Trypanosoma evansi* STIB805		+											
	*Trypanosoma grayi* ANR4		+											
	*Trypanosoma rangeli* SC58		+											
	*Trypanosoma vivax* Y486		+											
Phytomonas	*Phytomonas* Hart1		+											+
	*Phytomonas* EM1		+											+
Strigomonadinae	*Strigomonas culicis*		+							+			+	+
	*Angomonas deanei*		+							+			+	+
Leishmaniinae	*Leptomonas pyrrhocoris*		+							+			+	+
	*Crithidia fasciculata* CfCl		+					+		+			+	+
	*Leishmania braziliensis* MHOMBR75M2904		+					+		+			+	+
	*Leishmania donovani* BPK282A1		+					+		+			+	+
	*Leishmania infantum* JPCM5		+					+		+			+	+
	*Leishmania major* Friedlin		+					+		+			+	+
	*Leishmania mexicana* MHOMGT2001U1103		+					+		+			+	+
	*Leishmania tarentolae* ParrotTarII		+					+		+			+	+
Amoebozoa	*Paramoeba pemaquidensis* (host)	+	+			+		+	+		+	+		
	*Neoparamoeba aesturina* (host)	+	+			+	+	+	+	+	+			+
	*Paramoeba atlantica* (host)	+	+			+		+	+	+	+	+		

A ‘+’ indicates the presence of this enzyme. A ‘*’ indicates uncertainty associated with incomplete genomic or transcriptomic data. Blanks indicate protein absence or not detected

### Subcellular localization of the heme pathway in *Perkinsela* spp.

As shown in Table 1, several *Perkinsela* spp. appear to lack only one enzyme of the heme biosynthetic pathway (UROS), making it the most complete set identified for a kinetoplastid thus far. Other kinetoplastids have only a partial pathway or have lost the capacity to synthesize heme entirely; presumably they obtain heme from their host or do not require it. Perhaps due to its incomplete nature (when present), the subcellular localization of the heme pathway in other Kinetoplastea is different from the classical heme pathway in other eukaryotes [25]. We predicted the localization of the putative heme synthesis enzymes in *Perkinsela* sp. and compared these data to what is known from other kinetoplastids. We also predicted the localization of the heme pathway in the host amoebae, and compared them to other amoebozoans (Additional file 1: Table S1). As expected, FeCH seems to be targeted to the mitochondrion of *Perkinsela* spp., as in other heterotrophic organisms and in the FeCH-containing trypanosomatids. In addition, the heme pathway in *Perkinsela* spp., as well as in their *Neoparamoeba/Paramoeba* hosts, is predicted to produce 5-amino-levulinate in the mitochondrion. However, CPOX/HemF and PPOX/HemY enzymes were not predicted to be targeted to the mitochondrion.

### Complex phylogenetic patterns for heme biosynthesis enzymes in *Perkinsela* spp.

**Fig. 3:**
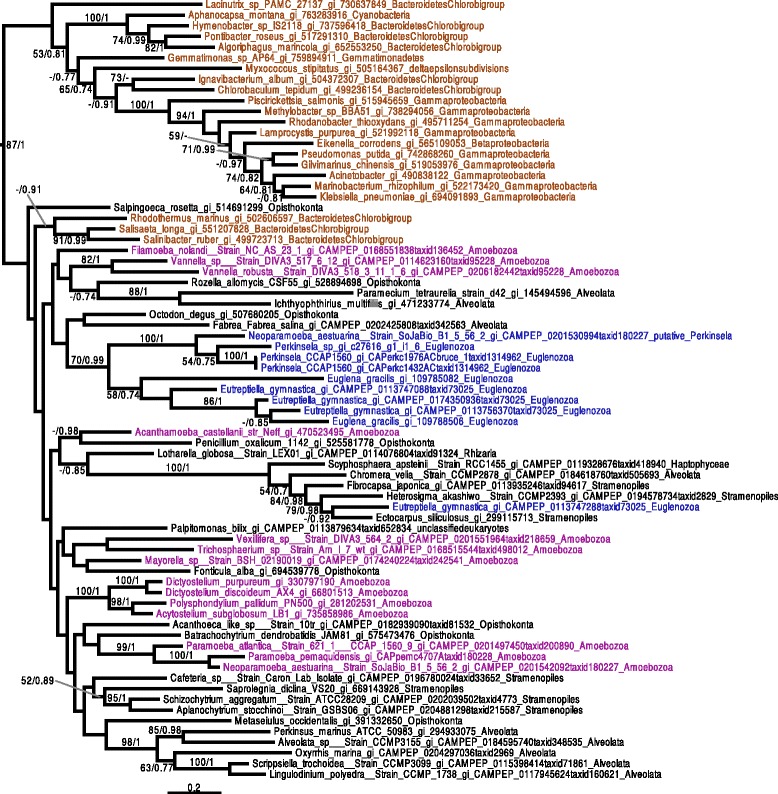
UROD phylogenetic tree. The tree shown is the consensus tree obtained with Phylobayes 4.1 with ML boostrap values (*left*) and Bayesian posterior probabilities (*right*) mapped onto the nodes. Bootstrap values >50 % are shown, while only posterior probabilities >0.6 are shown. The tree is rooted with the proteobacterial sequences, as in Kořený and Oborník [67]. Sequences are colored according to their taxonomic affiliation: Amoebozoa are in purple, Euglenozoa are in blue, other Eukaryota are in black, and Bacteria are brown. A trio of sequences from the euglenozoan* Eutreptiella gymnastica*, as well as two *Euglena gracilis* sequences, group with Prokinetoplastina with a bootstrap value of 70 % and a posterior probability of 0.99. Another *E. gymnastica* sequence branches elsewhere in the tree. The scale bar shows the inferred number of amino acid substitutions per site

**Fig. 4:**
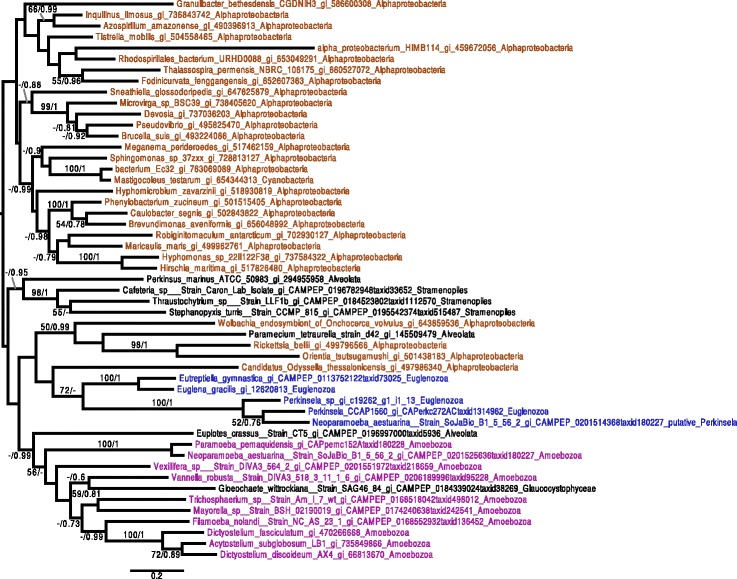
ALAS phylogenetic tree. The tree shown is the consensus tree obtained with Phylobayes 4.1 with ML boostrap values (*left*) and bayesian posterior probabilities (*right*) mapped onto the nodes. Bootstrap values >50 % are shown, while only posterior probabilities >0.6 are shown. Groups are color-coded according to taxonomy: Amoebozoa are in purple, Euglenozoa are in blue, other Eukaryota are in black, and Bacteria are brown. In this tree the Prokinetoplastina sequences branch together with *Euglena gracilis* and *Eutreptiella gymnastica* with a bootstrap value of 72 %. The scale bar shows the inferred number of amino acid substitutions per site

Given the patchy distribution of heme pathway enzymes in Kinetoplastea, we performed phylogenetic analyses for the complete set of *Perkinsela* spp. enzymes predicted to be involved in this pathway in an attempt to infer their evolutionary history. For two enzymes, UROD (Fig. 3) and ALAS (Fig. 4), the *Perkinsela* spp. homologs form a monophyletic clade with their counterparts in organisms to which they are known to be related, i.e., *Euglena* and/or *Eutreptiella*. Statistical support for this monophyletic relationship is reasonably strong in the case of UROD (bootstrap (BS) and posterior probabilities (PP) of 70 % and 0.99, respectively), but weak for ALAS. In both cases, homologues can be found in virtually all major eukaryotic groups and they seem to form a monophyletic clade (although the backbone of the trees is poorly resolved). This suggests that UROD and ALAS were likely present in the eukaryotic common ancestor and have been vertically inherited in *Perkinsela* spp. In addition, the eukaryotic ALAS homologues branch within alpha-proteobacteria, strongly suggesting a mitochondrial origin.

**Fig. 5:**
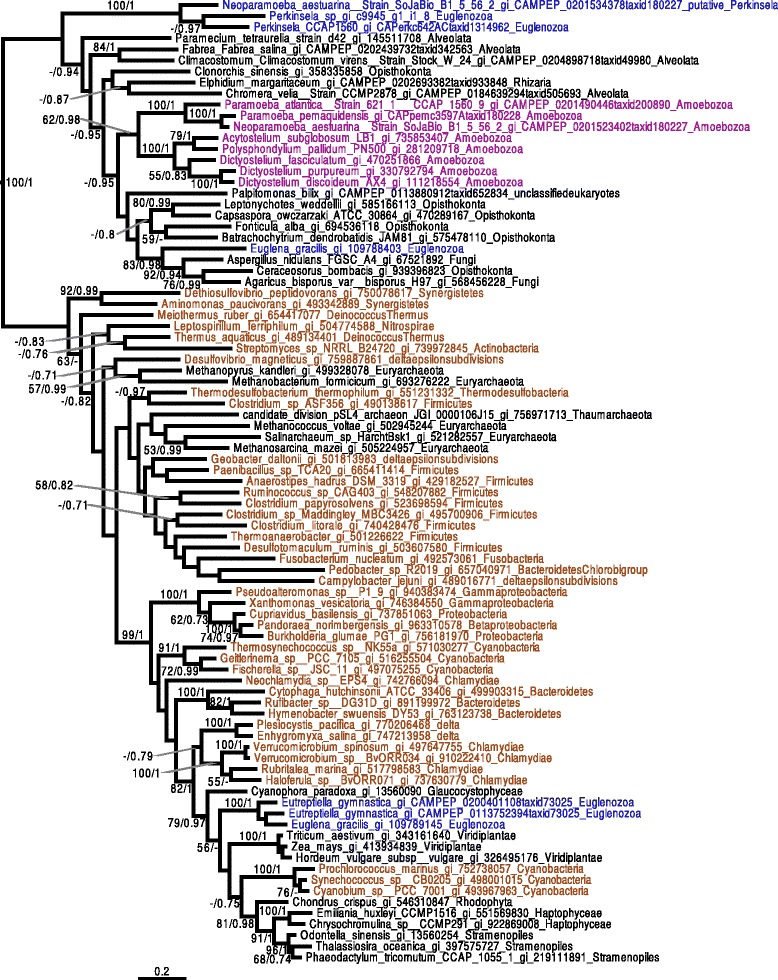
ALAD phylogenetic tree. The tree shown is the consensus tree obtained with Phylobayes 4.1 with ML boostrap values (*left*) and Bayesian posterior probabilities (*right*) mapped onto the nodes. Bootstrap values >50 % are shown, while only posterior probabilities >0.6 are shown. The tree is rooted with the distant group composed of bacteria. Color-coding: purple = Amoebozoa, blue = Euglenozoa, other eukaryotes and Archaea = black, Bacteria = brown. The Prokinetoplastina sequences branch at the base of a clade of eukaryotic homologs from Opisthokonta, Alveolata, Rhizaria and Amoebozoa. The scale bar shows the inferred number of amino acid substitutions per site

**Fig. 6:**
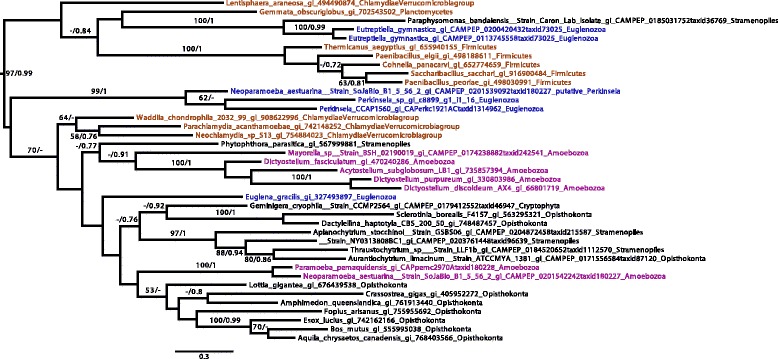
PPOX/HemY phylogenetic tree. The tree shown is the consensus tree obtained with Phylobayes 4.1 with ML boostrap values (*left*) and Bayesian posterior probabilities (*right*) mapped onto the nodes. Bootstrap values >50 % are shown, while only posterior probabilities >0.6 are shown. The tree is rooted with the distant group composed of Firmicutes, Planctomycetes and Lentisphaerae. Sequences are color-coded as follows: Amoebozoa are in purple, Euglenozoa are in blue, other eukaryotes are black, and Bacteria are brown. *Eutreptiella gymnastica*, *Euglena gracilis*, *Paraphysomonas bandaisensis* (all plastid-bearing organisms) and *Perkinsela* spp. sequences occupy distinct positions in the tree relative to other eukaryotes and bacterial homologs. The scale bar shows the inferred number of amino acid substitutions per site

**Fig. 7:**
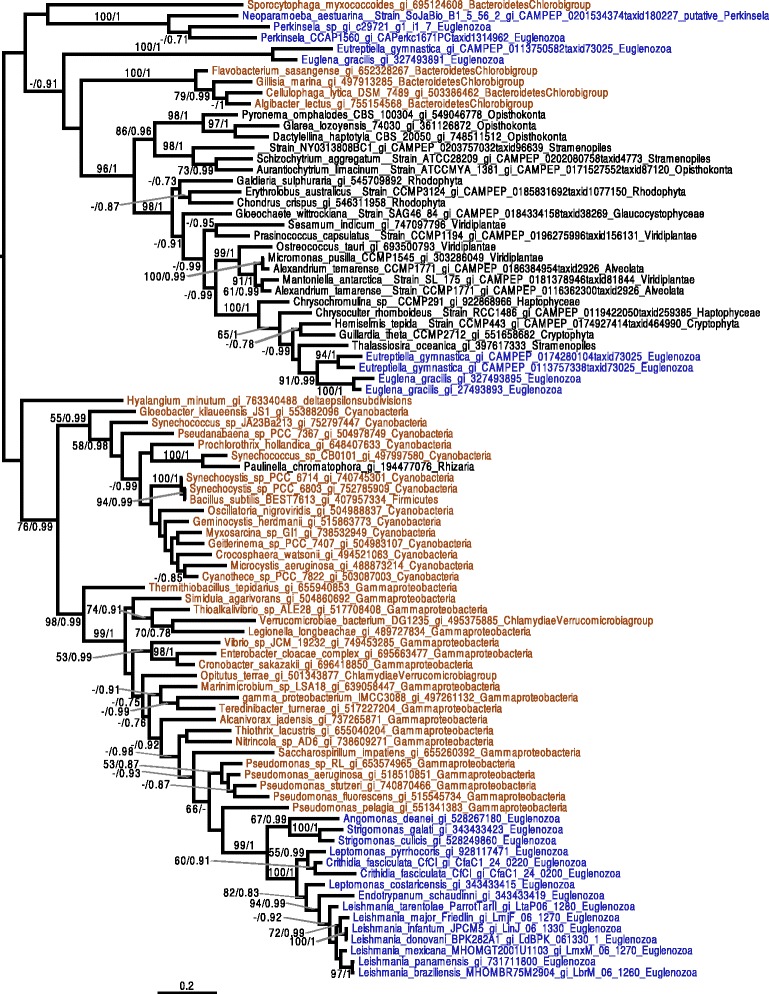
CPOX/HemF phylogenetic tree. The tree shown is the consensus tree obtained with Phylobayes 4.1 with ML boostrap values (*left*) and Bayesian posterior probabilities (*right*) mapped onto the nodes. Bootstrap values >50 % are shown, while only posterior probabilities >0.6 are shown. Groups are colored depending on their taxonomic group: Euglenozoa are in blue, other Eukaryotes are in black while Bacteria are colored brown. The *Perkinsela* spp. sequences group near the other eukaryotic sequences, albeit intermingled with bacterial sequences. The Leishmaniinae/Strigomonadinae sequences are nested within Gammaproteobacteria. The scale bar shows the inferred number of amino acid substitutions per site

In phylogenies of ALAD (Fig. 5), PBGD (Additional file 5: Figure S2.1), and PPOX/HemY (Fig. 6), *Perkinsela* spp. proteins generally branched amongst eukaryotic sequences, with bacterial sequences either being more distantly related or patchily distributed (see, e.g., Chlamydia homologs in the PPOX/HemY tree (Fig. 6)). In addition, PPOX/HemY and ALAD homologs found in *Perkinsela* spp. form a clade near the base of eukaryotes. In the case of CPOX/HemF (Fig. 7), while the *Perkinsela* spp. sequences are close to eukaryotes, the tree is complicated by the fact that the main eukaryotic clade contains sequences from Bacteroidetes. It is thus not possible to make definitive statements about the evolutionary origin of this gene in *Perkinsela* spp., although it seems likely that it represents the canonical eukaryotic CPOX. Our preliminary phylogenetic analyses of FeCH were suggestive of several lateral gene transfer (LGT) events in the history of this enzyme in Kinetoplastea (Additional file 5: Figures S2.2 and S2.4). However, the extremely large number of bacterial homologs available for this enzyme made comprehensive analyses difficult, and we suspected that the divergent nature of certain clades within the global FeCH tree were introducing long branch attraction artifacts. As described below, we thus systematically analyzed sub-sections of the FeCH tree in order to better understand the evolutionary history of this enzyme in the kinetoplastids.

### Lateral gene transfer and the evolution of heme biosynthetic enzymes in Kinetoplastea

Kořenỳ et al. proposed that heme enzymes in trypanosomatids have been acquired by LGT from various sources [25]. Indeed, the trypanosomatids are thought to have lost the heme pathway early in the evolution of the group [22], and while the Leishmaniinae/Strigomonadinae probably acquired genes for CPOX/HemF, PPOX/HemG and FeCH by LGT, the bodonids appear to have acquired FeCH separately, as have members of the genus *Phytomonas* [25].

Our results provide further support for the likelihood of LGT events from Gammaproteobacteria to an ancestor of Leishmaniinae and Strigomonadinae for CPOX/HemF (Fig. 7) and PPOX/HemG (Additional file 5: Figure S2.3). In addition, the FeCH phylogeny for these organisms suggests that Leishmaniinae and Strigomonadinae acquired the gene by LGT either from Gammaproteobacteria or from Firmicutes (Additional file 5: Figure S2.5). In the case of *Parabodo caudatus*, the FeCH homolog appears to be derived by LGT from Gammaproteobacteria (Additional file 5: Figure S2.6). The phylogeny of FeCH homologs in *Phytomonas* species is complicated by their divergent nature, but they nevertheless seem to have a different origin from their counterparts in the other Kinetoplastea discussed above (Additional file 5: Figure S2.7). However, FeCH enzymes in *Phytomonas* spp. appear to share recent common ancestry with FeCH homologs found in *Leptomonas* species.

### UROS and FeCH enzymes in Kinetoplastea: ancestral or recent acquisitions from bacteria?

**Fig. 8:**
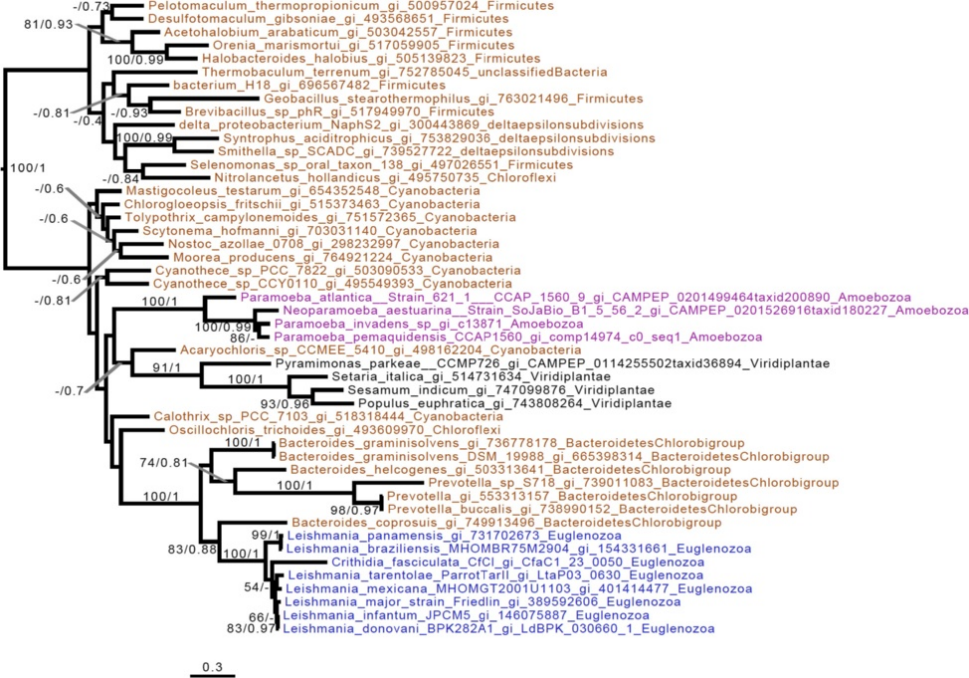
UROS phylogenetic tree. The tree shown is the consensus tree obtained with Phylobayes 4.1 with ML bootstrap values (*left*) and Bayesian posterior probabilities (*right*) mapped onto the nodes. Bootstrap values >50 % are shown, while only posterior probabilities >0.6 are shown. The tree is rooted with a distant bacterial group composed of Firmicutes/Proteobacteria/Chloroflexi. Taxonomic labels are as follows: Bacteria are brown, Euglenozoa are in blue, Amoebozoa are in purple, and other eukaryotes are in black. The sequences of the kinetoplastid genus *Leishmania* branch with a group of Bacteroidetes, which belong itself to a bigger group composed of cyanobacteria, Viridiplantae, and the *Paramoeba* spp. *Perkinsela* spp. sequences are not included, as no UROS genes were detected. The scale bar shows the inferred number of amino acid substitutions per site

While we were unable to detect any UROS related enzyme genes in the genome of the *Perkinsela* sp. within *P. pemaquidensis*, we found two different UROS gene candidates in *Paramoeba* spp., which, due to the presence of several introns, have both been assigned to be of host origin. The first UROS enzyme (KU609032) – the one included in the phylogenetic tree (Fig. 8) – shows a high degree of primary sequence conservation, and possesses homologs in all *Paramoeba* spp. included in this study (Table 1 and Additional file 4: Table. S3). The amino acid sequence of the other UROS candidate (KU609033), which was detected via HMMER search, is highly divergent; no phylogenetic trees could be constructed with this sequence. It is thus unclear whether this second host UROS-like enzyme functions in the heme pathway at all. In addition, we detected a putative UROS enzyme in Leishmaniinae, and carried out the first investigation of its evolutionary history. In our phylogenetic analysis, the Leishmaniinae and other eukaryotes, including the *Paramoeba* spp. group (i.e., the hosts of *Perkinsela* spp.), branch somewhat close to one another (Fig. 8), with the sequences from *Paramoeba* spp. showing affiliations to cyanobacterial homologs. *Leishmania* shows affiliations with a restricted group of Bacteroidetes. In an attempt to better understand the evolution of the *Leishmania* sequences, we removed the divergent bacterial group and reconstructed the tree. The resulting phylogeny (Additional file 5: Figure S2.8) still does not reveal any obvious link between Leishmaniinae and other eukaryotes. However, since the other eukaryotic sequences (*Paramoeba* spp. and Viridiplantae) found in this part of the UROS tree are separated by different bacteria, it seems reasonable to assume that the putative UROS gene of Leishmaniinae was acquired by LGT from bacteria.

As noted above, the FeCH enzyme of Prokinetoplastina is of ambiguous origin. In phylogenetic trees *Perkinsela* spp. homologs do not branch with those of Euglenozoa or Eukaryota (Additional file 5: Figures S2.9 and S2.10), although *Perkinsela* spp. and *Euglena gracilis* homologs are both nested within a restricted Bacteroidetes clade that could suggest lateral acquisition from this bacterial group. Strictly speaking, and despite the apparent rarity of LGT eukaryotes-to-prokaryotes [68], transfer from an early euglenozoan to Bacteroidetes cannot be excluded, and it is formally possible that the FeCH found in both *Euglena gracilis* and Prokinetoplastina represents the ancestral state for Euglenozoa.

## Discussion

### Evolution of Kinetoplastea

A wealth of genome and transcriptome data is now available from members of the Kinetoplastea. Recent studies on the evolution of this group [10, 69], together with the new data presented here, support a robust phylogeny with the Prokinetoplastina at the root of the Kinetoplastea. In addition, we show that the group formed by Leishmaniinae, Strigomonadinae and *Phytomonas* is robust (Fig. 2 and Additional file 3: Figure S1.1 and Table S1.2), as suggested in prior studies [1, 69]. Moreover, this group, called the LSP group in Fig. 2, is of particular interest from the perspective of heme biosynthetic enzymes in kinetoplastids, and the extent to which these organisms have lost or acquired heme pathway genes throughout their evolutionary history. We were unable to clearly resolve the relative branching order of *Phytomonas* spp., Leishmaniinae and Strigomonadinae. Maximum likelihood trees suggested a grouping of Leishmaniinae and Strigomonadinae, while Bayesian trees grouped Leishmaniinae and *Phytomonas* spp. together. However, these topological differences could be due to the different evolutionary models employed, as suggested by Brown et al. [70]. To test if this was the case, we built phylogenetic trees in which the evolutionary models and methods (i.e., maximum likelihood and Bayesian inference) were swapped. When the maximum likelihood analysis was performed with empirical profile mixture models, and the Bayesian approach was used with the LG model, the topologies corresponding to the original models were found in both cases, suggesting that it was the evolutionary model, not the tree building method, that was influencing the results (Additional file 3: Figure S1.3). In addition, since the empirical profile mixture models are considered to be more robust [71, 72] and our topological test with the LG4X model could not reject the close relationship between *Phytomonas* and Leishmaniinae (Additional file 3: Table S1.2), we consider a specific relationship between these two groups to the exclusion of Strigomonadinae to be most likely.

### Heme biosynthetic pathway evolution in *Perkinsela* spp.

We have shown that the *Perkinsela* spp. enzymes ALAS and UROD share recent common ancestry with those of euglenids, to which these endosymbionts are closely related [73, 74], and also that for ALAD, PBGD, CPOX/HemF, and PPOX/HemY enzymes, the *Perkinsela* spp. sequences generally branch within a eukaryotic clade represented by most or all of the major eukaryotic lineages. This is consistent with the hypothesis that the heme pathway in *Perkinsela* spp. represents, at least in part, the ancestral pathway present in Kinetoplastea. Nevertheless, there is growing evidence supporting an important role for LGT in eukaryotes (for review see [75]), one that is particular well described in unicellular organisms [76–78]. A role for LGT in the case of *Perkinsela* spp. heme biosynthesis should thus not be dismissed.

On one hand, the ALAS and UROD enzymes of *Perkinsela* spp. were most likely inherited vertically, since they seem to share recent common ancestry with euglenozoan enzymes. On the other hand, the genes for ALAD, PBGD, CPOX/HemF, and PPOX/HemY could represent more recent acquisitions by LGT, perhaps from a eukaryotic donor. However, homologs of CPOX/HemF, PPOX/HemY and ALAD are widely distributed amongst eukaryotes and they seem to form a monophyletic clade. In addition, the position of the Prokinetoplastina at the root of eukaryotes seems to not argue in favor of recent acquisitions by LGT, particularly in light of recent articles that place excavates at the root of the eukaryotic tree [79–81].

In the case of FeCH, the *Perkinsela* spp. gene was either acquired by LGT from Bacteroidetes or present in a common ancestor shared with Euglenozoa. Under the scenario of it being present ancestrally, the presence of Bacteroidetes homologs in this part of the tree could mean LGT from Euglenozoa to Bacteroidetes (Additional file 5: Figures S2.9 and S2.10). It seems reasonable to speculate that poor taxonomic sampling for this gene across the full breadth of the eukaryotic tree is contributing to our inability to distinguish between a lateral or vertical evolutionary history for this enzyme in Kinetoplastea.

The only enzyme of the heme biosynthetic pathway that appears to be absent from the *Perkinsela* sp. genome is UROS. However, as we have identified a potential UROS enzyme in Leishmaniinae (see Table 1 and Fig. 8), we cannot definitively reject the ancestral nature of this protein. Indeed, the fact that the Leishmaniinae homologs branch relatively close to UROS from other eukaryotes (including the *Paramoeba* sp. host) is consistent with this possibility. Alternatively, the Leishmaniinae and/or other eukaryotes could have acquired the putative UROS gene by LGT from bacteria independently.

Interestingly, we found two potential UROS candidates in the *Paramoeba pemaquidensis* nuclear genome, which could indicate two different UROS functions in the host amoeba. However, the level of sequence conservation of one of the two putative host UROS enzymes (KU609033; not identifiable by BLASTp, only through HMMER searches) is so low that it was not possible to include it in our phylogenetic analysis. We are thus presently only confident in the presence of one UROS enzyme in the *Paramoeba* species we investigated (KU609032 in *P. pemaquidensis*). In any case, the apparent absence of a gene encoding UROS in the *Perkinsela* sp. genome, and the presence of at least one UROS in *Paramoeba* spp., raises the possibility of metabolic interdependency between the two cells. It is conceivable that a host-encoded UROS protein and/or metabolic intermediates make their way to the kinetoplastid endosymbiont in order to compensate for the gap in the heme biosynthetic pathway of the endosymbiont. Further research is needed to confirm or refute this hypothesis (see below).

### Several independent lateral acquisitions of the heme biosynthetic pathway in Leishmaniinae/Strigomononadinae, *Phytomonas* and *Parabodo*

The discovery of a near-full heme pathway in a basal kinetoplastid allows us to assess loss, acquisition by LGT, or retention of the heme pathway in Kinetoplastea. Indeed, recent studies suggest that CPOX/HemF and PPOX/HemG were acquired by LGTs in Leishmaniinae, while FeCH appears to have been acquired three different times in kinetoplastids: in Leishmaniinae/Strigomononadinae, in *Phytomonas* and also in Bodonida [22, 25]. Here, with the inclusion of new sequences from basal-branching kinetoplastids and other Euglenozoa, LGT of CPOX/HemF and PPOX/HemG from Gammaproteobacteria to Leishmaniinae/Strigomononadinae can be inferred (Fig. 7 and Additional file 5: Figure S2.3), as well as LGT of FeCH from Firmicutes or Gammaproteobacteria to Leishmaniinae/Strigomonadinae (Additional file 5: Figure S2.5). However, the FeCH present in *Phytomonas* groups with the Leishmaniinae genus *Leptomonas*, and could be the product of a separate LGT event (Additional file 5: Figures S2.2 and S2.7). Altogether these results strongly support the hypothesis that heme biosynthetic enzymes in Leishmaniinae/Strigomonadinae, *Phytomonas*/*Leptomonas* and *Parabodo* were acquired by LGT.

### Heme pathway evolution in Kinetoplastea

**Fig. 9:**
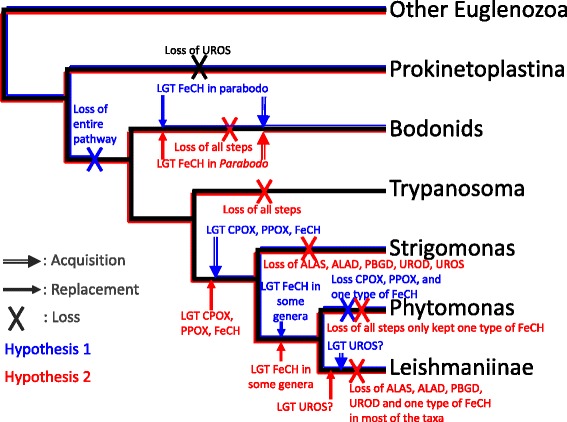
Hypotheses for heme biosynthesis pathway loss and acquisition in Kinetoplastea. (i) Under Hypothesis 1, the heme pathway was lost early in Kinetoplastea evolution, after the split with Prokinetoplastina, and before the emergence of bodonids. Subsequently, Strigomonadinae, Leishmaniinae and *Phytomonas* spp. acquired FeCH genes several times from different sources, while the Leishmaniinae and Strigomonadinae group obtained CPOX and HemG from a proteobacterium, and Leishmaniinae gained UROS from a Bacteroidetes. Some organisms in the bodonids acquired an FeCH gene. (ii) Under Hypothesis 2, the heme pathway was lost separately in bodonids, *Trypanosoma*, and lost in part in *Phytomonas* spp., and Leishmaniinae/Strigomonadinae. Subsequently, *Phytomonas* spp*.,* Leishmaniinae, Strigomonadinae replaced part of their heme metabolism by the lateral acquisition of enzymes from different sources

Based on our analyses, we considered three different scenarios for the evolution of the heme biosynthetic pathway in Kinetoplastea (Fig. 9). The first possibility is that the heme pathway was present ancestrally but lost early in the evolution of Kinetoplastea, after the split with Prokinetoplastina, and before the emergence of bodonids. Subsequently, Strigomonadinae, Leishmaniinae and *Phytomonas* spp. acquired FeCH several times from different sources, while the Leishmaniinae and Strigomonadinae taxa obtained CPOX/HemF and PPOX/HemG from a proteobacterium, and Leishmaniinae alone gained UROS from a Bacteroidetes. Some organisms within the bodonids gained FeCH, as proposed by Kořenỳ et al. [22]. A second hypothesis is that the heme pathway was lost separately in bodonids, *Trypanosoma*, and lost in part in *Phytomonas* spp., and Leishmaniinae/Strigomonadinae. Subsequently, *Phytomonas* spp*.,* Leishmaniinae, Strigomonadinae replaced part of their metabolism by LGT from different sources. Those replacements might have been related to their adaptation to a parasitic lifestyle, as is the case of the PPOX/HemG enzyme that possesses the dual ability to act under anaerobic and aerobic conditions [36]. Finally, a third hypothesis revolves around the notion that LGT has not been a significant factor in eukaryotic evolution in general and the evolution of the heme pathway in particular. This scenario would necessitate the existence of a gene-rich organism at the root of Kinetoplastea and eukaryotes, where the presence/absence of the different components of the heme pathway in modern-day organisms is the result of differential gene loss. This latter hypothesis is in line with recent proposals for the rarity of LGT in eukaryotes [82, 83].

Given that the putative LGTs of CPOX/HemY and PPOX/HemG genes from Gammaproteobacteria to Leishmaniinae and Strigomonadinae are highly supported and no other eukaryotic homologs appear closely related (Fig. 7, Additional file 5: Figure 2.3), we consider the first two hypotheses to be more likely. Furthermore, we favor the second hypothesis over the first, for two reasons, first because shared common ancestry between the UROS enzymes of Leishmaniinae and other eukaryotes (including the *Paramoeba* spp. host and Viridiplantae) cannot be rejected outright (Fig. 8 and Additional file 5: Figure S2.8), and second, from a biochemical standpoint, gene replacement is perhaps more feasible. Indeed, it is easier to replace enzymes in an existing biochemical pathway than to acquire individual enzymes *de novo*, since there is no need for an organism to retain single enzymes from a pathway in isolation, particularly those catalyzing intermediate steps. Nevertheless, because of the exceedingly complicated phylogenies of several of the enzymes analyzed herein, it is difficult to determine which of these two scenarios is more likely. The individual phylogenies of the heme pathway proteins could in fact be explained by a combination of hypotheses one and two, e.g., an early loss of part of the heme pathway followed by differential losses of other enzymes in specific lineages.

### Presence/absence of key enzymes in *Paramoeba pemaquidensis* and its *Perkinsela* endosymbiont suggests an intimate link between these organisms

Given that seven of the eight ‘core’ heme pathway enzymes exist in *Perkinsela* spp., and most of the enzymes, like those of their hosts, appear to have ‘standard’ subcellular localizations (Additional file 1: Table S1), the endosymbiont and host heme pathways appear to operate mainly separately. However, the absence of a UROS gene in the *Perkinsela* sp. genome and the presence of at least one UROS in the *Paramoeba* sp. genome is suggestive of metabolic ‘cross-talk’. Could there be heme (intermediate) exchange between the two? Interestingly, heme transporters have recently been characterized in *Leishmania* [26, 84] and heme uptake in *Trypanosoma brucei* might be receptor-mediated [27, 85]. We searched for, but did not find, obvious homologs of the *Leishmania* and *Trypanosoma* heme transporters in the nuclear genome of *Perkinsela* sp. Two scenarios are possible: either the evolution of such heme transporters in an ancestor of kinetoplastids took place after the divergence of Prokinetoplastina, or the transporters were lost in Prokinetoplastina. Since the Prokinetoplastina sequences currently available belong to intracellular endosymbionts, it is possible that the related transporters have been lost as a result of reductive evolution.

While the heme biosynthetic pathway provides heme as a cofactor for metabolic processes such as oxidative phosphorylation, growing evidence indicates that some organisms can thrive without it [22, 86]. Reduction and outright loss of the pathway in several Kinetoplastea has been documented, e.g., in *Leishmania* and *Trypanosoma* [25], which makes the finding of a near-complete pathway in the kinetoplastid endosymbiont *Perkinsela* sp. all the more intriguing. Given its obligate intracellular lifestyle, it would not be surprising if it were capable of taking heme, or at least intermediates of the heme biosynthetic pathway, from its host. A common theme in cellular evolution is the reduction of pathways in organisms that live (endo) symbiotically [87, 88], particularly in cases where the host can produce necessary metabolites, as is presumably the case with the amoeba hosts of *Perkinsela* spp. Although only one heme pathway enzyme appears to be missing in *Perkinsela* spp., it is a key component and suggestive of host-endosymbiont metabolic dependency.

In addition, it is interesting to note that in the *Perkinsela* sp. endosymbiont, the reaction producing protoporphyrinogen IX is most likely catalized by CPOX/HemF, while the amoeba host possesses both CPOX/HemF, which acts in aerobic environments, and a CPOX/HemN homolog predicted to function anaerobically. Thus under anaerobic conditions, only the host might be capable of producing the required heme intermediates needed by both organisms. Indeed, even in the absence of specific transporters for heme or heme pathway intermediates, it is possible that non-specific mechanisms such as endocytosis might play a role in host-endosymbiont heme exchange. A better understanding of the metabolic and cell biological communication between host and endosymbiont will hopefully shed light on the nature of this unusual endosymbiotic relationship.

## Conclusions

Here we have explored the basal evolutionary position of Prokinetoplastina within Kinetoplastea, and provided the first evidence for the existence of a near-complete heme biosynthesis pathway in a member of the Kinetoplastea. Phylogenetic analyses suggest that a major portion of the heme pathway present in Prokinetoplastina (in particular, *Perkinsela* spp.) was ancestrally present. LGT also appears to have contributed heme biosynthetic genes after the various Kinetoplastea lineages diverged from one another, perhaps related to their adaptation to a parasitic lifestyle. The presence of an almost complete heme pathway in *Perkinsela* spp. provides an opportunity for future studies aimed at shedding light on the nature of the metabolic connections between *Perkinsela* and its *Paramoeba* host.

### Availability of data and materials

All data are available through NCBI. Materials (*Paramoeba* strains) are available on request and from the CCAP. Phylogenetic data are available in Dryad: https://datadryad.org/resource/doi:10.5061/dryad.664cp.2.

## Additional files

Additional file 1: Table S1. Table of localization predictions for proteins involved in heme metabolism in Kinetoplastea. (XLSX 15 kb) Additional file 2: Table S2. Table with accession numbers of proteins used for the Kinetoplastea phylogeny. (XLSX 12 kb) Additional file 3: Figure S1. Supplementary figures (phylogenetic trees and tables) for the Kinetoplastea phylogeny. (PDF 361 kb) Additional file 4: Table S3. Table with accession numbers of the proteins involved in the heme pathway, encoded in Kinetoplastea and the host Paramoeba/Neoparamoeba. (XLSX 72 kb)) Additional file 5: Figure S2. Supplementary phylogenetic trees of proteins involved in heme pathway, encoded in the different Kinetoplastea. (PDF 7181 kb)

## Abbreviations

ALAD: delta-aminolevulinic acid dehydratase
ALAS: 5-aminolevulinate synthase
BTS: bipartite targeting signal
CPOX/HemF: coproporphyrinogen III oxidase
CPOX/HemN: oxygen-independent coproporphyrinogen III oxidase
FeCH: ferrochelatase
GltX: glutamyl-tRNA synthetase
GSA-AT: glutamate-1-semialdehyde 2,1-aminomutase
GTR: glutamyl-tRNA reductase
LGT: lateral gene transfer
PBGD: porphobilinogen deaminase
PPOX/HemG: menaquinone-dependent protoporphyrinogen oxidase
PPOX/HemY: oxygen-dependent protoporphyrinogen oxidase
SP: signal peptide
TMD: transmembrane domain
UROD: uroporphyrinogen decarboxylase
UROS: uroporphyrinogen-III synthase
