# Supplementary material for: Heme pathway evolution in kinetoplastid protists
Source: BMC Evol Biol. 2016 May 18;16:109. doi: 10.1186/s12862-016-0664-6 (PMC4870792; doi:10.1186/s12862-016-0664-6)
Supplement: Additional file 3: Figure S1. — Supplementary figures (phylogenetic trees and tables) for the Kinetoplastea phylogeny. (PDF 361 kb) [file 12862_2016_664_MOESM3_ESM.pdf]

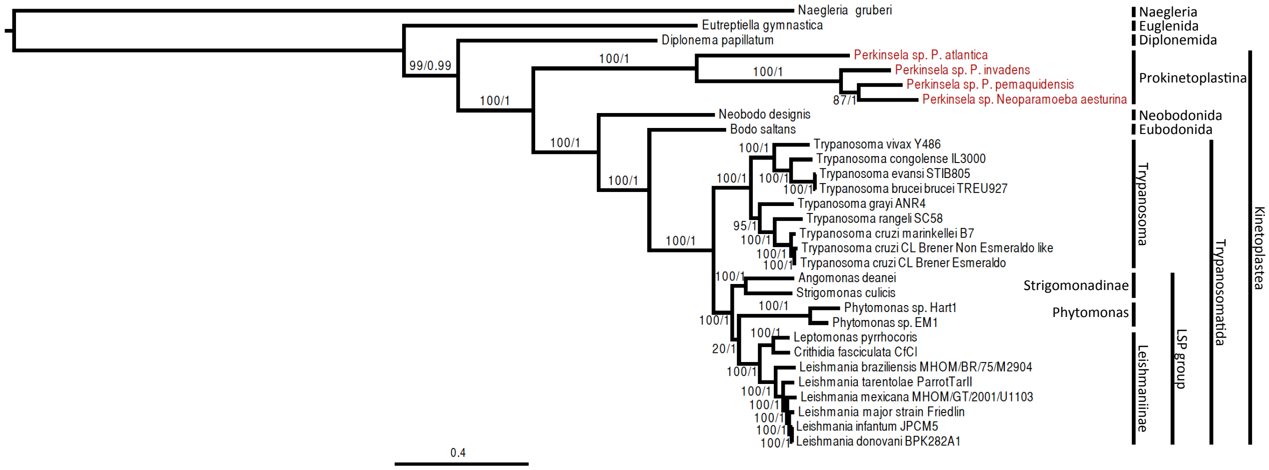

Figure S1.1: Phylogeny of Kinetoplastea based on a concatenation of 11 proteins. The tree was built using the C20 + Poisson model with Phylobayes 4.1. 1,000 bootstrap repetitions were performed using the LG4X model IQTREE and mapped onto the nodes (left) alongside posterior probabilities (right). The Prokinetoplastina, represented by *Perkinsela* spp. are highlighted red. Higher-level taxonomic classifications are indicated on the right for each group of organisms. The Kinetoplastea form a group with Diplonemida and Euglenida forming successively deeper branches when the heterolobosean *Naegleria gruberi* is used to root the tree. The Prokinetoplastina are a well-supported, early-branching group of Kinetoplastea. The bodonids branch as sister to the Trypanosomatida, while Leishmaniinae, *Phytomonas* and Strigomonadinae form a strongly supported, distinct group. The scale bar shows the inferred number of amino acid substitutions per site.

Table S1.2: Topology test results for the Kinetoplastea (Figure 1) using the LG4X model in RAxML. The groups tested are indicated on the right. For each test, the following information is provided: bp-RELL, p-KH, p-SH, c-ELW. A ‘+’ sign indicates that the topology is not rejected, while ‘-’ indicates rejection. Abbreviations: Ngr: *N. gruberi*, Egy: *E. gymnastica*, Dpa: *D. papillatum*, Nde: *N. designis*, Bsa: *B. saltans*, PhH: *Phytomonas* Hart1, PhE: *Phytomonas* EM1, Ade: *A. deanei*, Cfa: *C. fasciculata* CfCl, Lpy: *L. pyrrhocoris*, Scu: *S. culicis* , Lbr: *L. braziliensis* MHOMBR75M2904, Lta: *L. tarentolae* ParrotTarII, Lme: *L. Mexicana* MHOMGT2001U1103, Lma: *L. major* Friedlin, Ldo: *L. donovani* BPK282A1, Lin: *L. infantum* JPCM5, Pea: *P. atlantica*, Pei: *Perkinsela* from *Paramoeba invadens*, Nea: *N. aestuarina*, Pep: *Perkinsela* from *Paramoeba pemaquidensis*, Tgr: *T. grayi* ANR4, Tra: *T. rangeli* SC58, Tcm: *T. cruzi* marinkellei B7, TcBE: *T. cruzi* CL Brener Esmeraldo like, TcBN: *T. cruzi* CL Brener Non Esmeraldo like, Tvi: *T. vivax* Y486, Tco: *T. congolense* IL3000, TbT: *T. brucei* TREU927, TbL: *T. brucei* Lister 427, TbL: *T. brucei* gambiense DAL972, Tev: *T. evansi* STIB805.

Trees	logL	deltaL	bp-RELL	p-KH	p-SH	c-ELW	
**(Ngr,Egy,Dpa,(Nde,Bsa,((Pea,Pei,Nea,Pep),(PhE,PhH),Ade,Cfa,Lpy,Scu,Lbr,Lta,Lme,Lma,Ldo,Lin),((Tgr,Tra,Tcm,TcBE,TcBN),(Tvi,Tco,TbT,Tev))))**	-107771.796	772.869	0.0000 -	0.0000 -	0.0000 -	0.0000 -	Test Prokinetoplastina
**(Ngr,Egy,Dpa,(Nde,Bsa,((PhE,PhH),Ade,Cfa,Lpy,Scu,Lbr,Lta,Lme,Lma,Ldo,Lin),(((Pea,Pei,Nea,Pep),Tgr,Tra,Tcm,TcBE,TcBN),(Tvi,Tco,TbT,Tev))))**	-108059.426	1060.498	0.0000 -	0.0000 -	0.0000 -	0.0000 -	
**(Ngr,Egy,Dpa,(Nde,Bsa,((PhE,PhH),Ade,Cfa,Lpy,Scu,Lbr,Lta,Lme,Lma,Ldo,Lin),((Tgr,Tra,Tcm,TcBE,TcBN),((Pea,Pei,Nea,Pep),Tvi,Tco,TbT,Tev))))**	-108051.205	1052.278	0.0000 -	0.0000 -	0.0000 -	0.0000 -	
**(Ngr,Egy,Dpa,(Nde,Bsa,((PhE,PhH),Ade,Cfa,Lpy,Scu,Lbr,Lta,Lme,Lma,Ldo,Lin),((Pea,Pei,Nea,Pep),(Tgr,Tra,Tcm,TcBE,TcBN),(Tvi,Tco,TbT,Tev))))**	-107772.682	773.754	0.0000 -	0.0000 -	0.0000 -	0.0000 -	
**(Ngr,Egy,((Pea,Pei,Nea,Pep),(Dpa,(Nde,Bsa,((PhE,PhH),Ade,Cfa,Lpy,Scu,Lbr,Lta,Lme,Lma,Ldo,Lin),((Tgr,Tra,Tcm,TcBE,TcBN),(Tvi,Tco,TbT,Tev))))))**	-107026.713	27.786	0.0080 +	0.0420 -	0.8730 +	0.0022 +	
**(Ngr,((Pea,Pei,Nea,Pep),(Egy,Dpa,(Nde,Bsa,((PhE,PhH),Ade,Cfa,Lpy,Scu,Lbr,Lta,Lme,Lma,Ldo,Lin),((Tgr,Tra,Tcm,TcBE,TcBN),(Tvi,Tco,TbT,Tev))))))**	-107082.217	83.29	0.0000 -	0.0010 -	0.4000 +	0.0000 -	
**(Ngr,Egy,Dpa,(Nde,((Pea,Pei,Nea,Pep),Bsa,((PhE,PhH),Ade,Cfa,Lpy,Scu,Lbr,Lta,Lme,Lma,Ldo,Lin),((Tgr,Tra,Tcm,TcBE,TcBN),(Tvi,Tco,TbT,Tev)))))**	-107155.537	156.61	0.0000 -	0.0000 -	0.0490 -	0.0000 -	
**(Ngr,Egy,Dpa,((Pea,Pei,Nea,Pep),Nde,Bsa,((Ade,Scu),((PhE,PhH),(Cfa,Lpy,Lbr,Lta,Lme,Lma,Ldo,Lin))),((Tgr,Tra,Tcm,TcBE,TcBN),(Tvi,Tco,TbT,Tev))))**	-107028.707	29.779	0.0160 +	0.1180 +	0.8590 +	0.0006 +	Test Phytomonas
**(Ngr,Egy,Dpa,((Pea,Pei,Nea,Pep),((PhE,PhH),Nde,Bsa),((Ade,Scu),(Cfa,Lpy,Lbr,Lta,Lme,Lma,Ldo,Lin)),((Tgr,Tra,Tcm,TcBE,TcBN),(Tvi,Tco,TbT,Tev))))**	-107618.093	619.166	0.0000 -	0.0000 -	0.0000 -	0.0000 -	
**(Ngr,Egy,Dpa,((Pea,Pei,Nea,Pep),Nde,(Bsa,PhE,PhH),((Ade,Scu),(Cfa,Lpy,Lbr,Lta,Lme,Lma,Ldo,Lin)),((Tgr,Tra,Tcm,TcBE,TcBN),(Tvi,Tco,TbT,Tev))))**	-107449.631	450.704	0.0000 -	0.0000 -	0.0000 -	0.0000 -	
**(Ngr,Egy,Dpa,((Pea,Pei,Nea,Pep),(Nde,PhE,PhH),Bsa,((Ade,Scu),(Cfa,Lpy,Lbr,Lta,Lme,Lma,Ldo,Lin)),((Tgr,Tra,Tcm,TcBE,TcBN),(Tvi,Tco,TbT,Tev))))**	-107736.643	737.715	0.0000 -	0.0000 -	0.0000 -	0.0000 -	
**(Ngr,Egy,Dpa,((Pea,Pei,Nea,Pep),Nde,Bsa,((Ade,Scu),(Cfa,Lpy,Lbr,Lta,Lme,Lma,Ldo,Lin)),((PhE,PhH),(Tgr,Tra,Tcm,TcBE,TcBN),(Tvi,Tco,TbT,Tev))))**	-107149.315	150.388	0.0000 -	0.0000 -	0.0550 +	0.0000 -	
**(Ngr,Egy,Dpa,(((PhE,PhH),Pea,Pei,Nea,Pep),Nde,Bsa,((Ade,Scu),(Cfa,Lpy,Lbr,Lta,Lme,Lma,Ldo,Lin)),((Tgr,Tra,Tcm,TcBE,TcBN),(Tvi,Tco,TbT,Tev))))**	-107879.349	880.422	0.0000 -	0.0000 -	0.0000 -	0.0000 -	
**(Ngr,Egy,Dpa,((Pea,Pei,Nea,Pep),Nde,Bsa,((PhE,PhH),(Cfa,Lpy,Lbr,Lta,Lme,Lma,Ldo,Lin)),((Ade,Scu),(Tgr,Tra,Tcm,TcBE,TcBN),(Tvi,Tco,TbT,Tev))))**	-107223.188	224.261	0.0000 -	0.0000 -	0.0040 -	0.0000 -	Test Strigomanidae
**(Ngr,Egy,Dpa,(((Ade,Scu),Pea,Pei,Nea,Pep),Nde,Bsa,((PhE,PhH),(Cfa,Lpy,Lbr,Lta,Lme,Lma,Ldo,Lin)),((Tgr,Tra,Tcm,TcBE,TcBN),(Tvi,Tco,TbT,Tev))))**	-108017.616	1018.688	0.0000 -	0.0000 -	0.0000 -	0.0000 -	
**(Ngr,Egy,Dpa,((Pea,Pei,Nea,Pep),Nde,((Ade,Scu),Bsa),((PhE,PhH),(Cfa,Lpy,Lbr,Lta,Lme,Lma,Ldo,Lin)),((Tgr,Tra,Tcm,TcBE,TcBN),(Tvi,Tco,TbT,Tev))))**	-107591.189	592.261	0.0000 -	0.0000 -	0.0000 -	0.0000 -	
**(Ngr,Egy,Dpa,((Pea,Pei,Nea,Pep),((Ade,Scu),Nde),Bsa,((PhE,PhH),(Cfa,Lpy,Lbr,Lta,Lme,Lma,Ldo,Lin)),((Tgr,Tra,Tcm,TcBE,TcBN),(Tvi,Tco,TbT,Tev))))**	-107875.603	876.676	0.0000 -	0.0000 -	0.0000 -	0.0000 -	
**(Ngr,Egy,Dpa,((Pea,Pei,Nea,Pep),(Cfa,Lpy,Lbr,Lta,Lme,Lma,Ldo,Lin),(Nde,Bsa,(Ade,Scu),((PhE,PhH),((Tgr,Tra,Tcm,TcBE,TcBN),(Tvi,Tco,TbT,Tev))))))**	-107900.434	901.507	0.0000 -	0.0000 -	0.0000 -	0.0000 -	Test Leishmani-inae
**(Ngr,Egy,Dpa,(Pea,Pei,Nea,Pep),Nde,Bsa,((PhE,PhH),Ade,Scu),((Lpy,Cfa,Lbr,Lta,Lme,Lma,Ldo,Lin),(Tgr,Tra,Tcm,TcBE,TcBN),(Tvi,Tco,TbT,Tev)))**	-107274.14	275.213	0.0000 -	0.0000 -	0.0020 -	0.0000 -	
**(Ngr,Egy,Dpa,((Pea,Pei,Nea,Pep),Nde,Bsa,(Lpy,Cfa,Lbr,Lta,Lme,Lma,Ldo,Lin),(((PhE,PhH),Ade,Scu),((Tgr,Tra,Tcm,TcBE,TcBN),(Tvi,Tco,TbT,Tev)))))**	-107275.172	276.245	0.0000 -	0.0000 -	0.0000 -	0.0000 -	
**No constraint**	-106998.927	0	0.0240 +	0.5050 +	1.0000 +	0.0013 +	

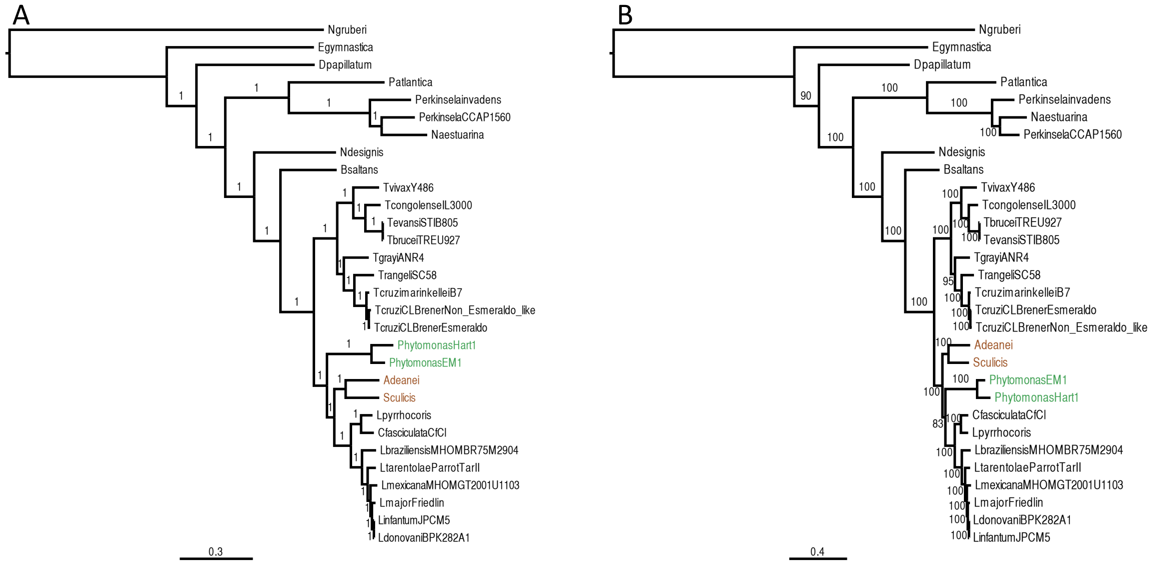

Figure S1.3: (A) Phylogenetic tree of Kinetoplastea and related lineages constructed using phylobayes with the LG model. (B) Phylogenetic tree using IQTREE and the ultrafast bootstrap method with the C20 model. The *Phytomonas* spp. is highlighted in green, while Strigomanidae is colored in brown. Scale bar indicates the inferred number of substitutions per amino acid site.

Table S1.4: Percentage of missing data for each organism in the alignment used to construct the Kinetoplastea evolution tree.

	Gaps
**A. deanei**	2.67%
**B. saltans**	25.10%
**C. fasciculata CfCl**	14.35%
**D. papillatum**	42.49%
**E. gymnastica**	12.27%
**L. braziliensis MHOMBR75M2904**	0.57%
**L. donovani BPK282A1**	0.53%
**L. infantum JPCM5**	0.53%
**L. major Friedlin**	0.53%
**L. mexicana MHOMGT2001U1103**	0.53%
**L. pyrrhocoris**	0.00%
**L. tarentolae Parrot TarII**	0.00%
**N. aestuarina**	10.73%
**N. designis**	9.53%
**N. gruberi**	24.09%
**P. atlantica**	47.63%
**Perkinsela CCAP1560/4**	1.11%
**Perkinsela invadens**	0.89%
**Phytomonas EM1**	15.36%
**Phytomonas Hart1**	13.77%
**S. culicis**	5.97%
**T. brucei TREU927**	0.04%
**T. congolense IL 3000**	20.71%
**T. cruzi CL Brener Esmeraldo**	22.04%
**T. cruzi CL Brener Non Esmeraldo like**	15.89%
**T. cruzi marinkellei B7**	7.55%
**T. evansi STIB805**	0.04%
**T. grayi ANR4**	2.89%
**T. rangeli SC58**	7.91%
**T. vivax Y486**	3.42%
