# Supplementary material for: Heme pathway evolution in kinetoplastid protists
Source: BMC Evol Biol. 2016 May 18;16:109. doi: 10.1186/s12862-016-0664-6 (PMC4870792; doi:10.1186/s12862-016-0664-6)
Supplement: Additional file 5: Figure S2. — Supplementary phylogenetic trees of proteins involved in heme pathway, encoded in the different Kinetoplastea. (PDF 7181 kb) [file 12862_2016_664_MOESM5_ESM.pdf]

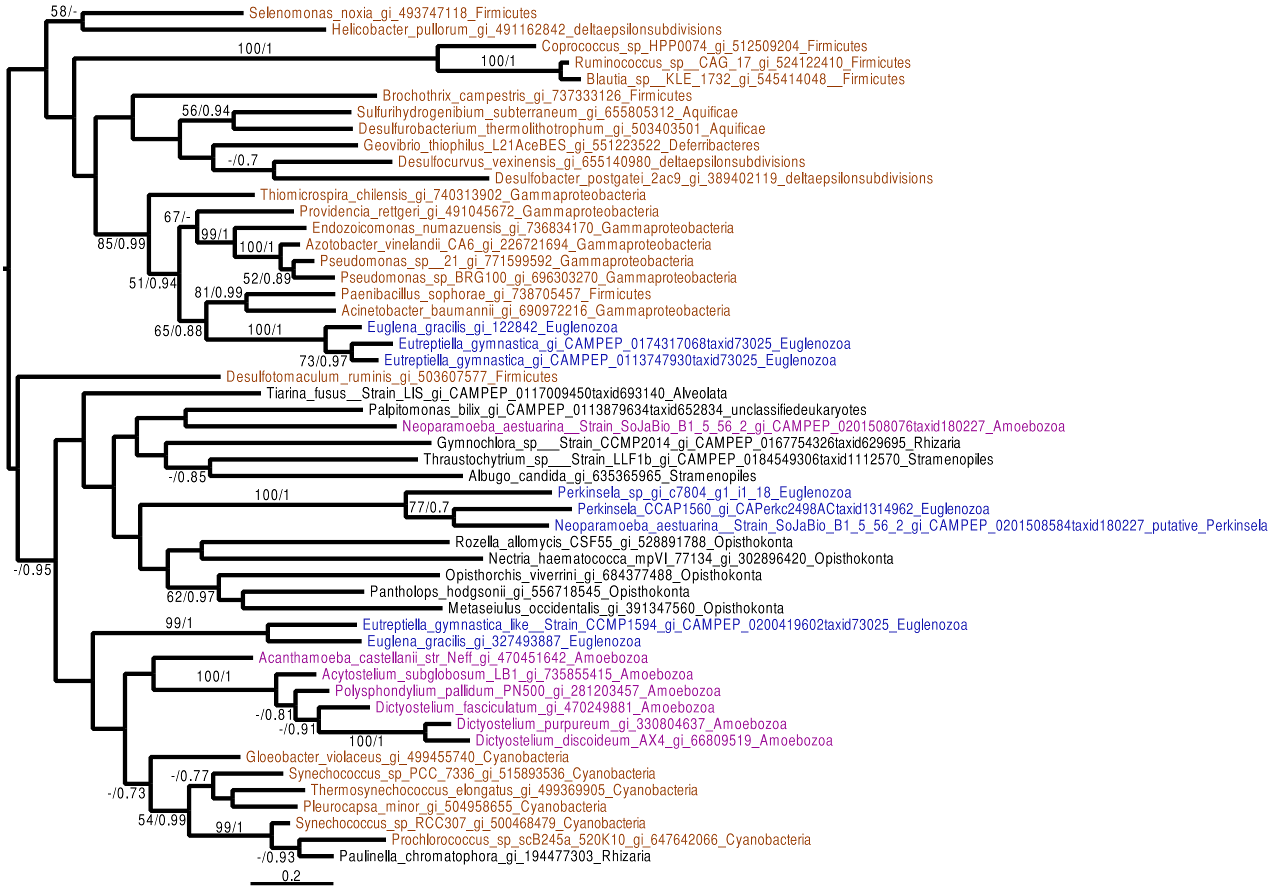

Figure S2.1: PBGD phylogenetic tree. The tree shown is the consensus tree obtained with Phylobayes 4.1 with ML boostrap values (left) and bayesian posterior probabilities (right) mapped onto the nodes. Bootstrap values >50% are shown, while only posterior probabilities >0.6 are shown. Groups are colored according to their taxonomic affiliation: Amoebozoa are in purple, Euglenozoa are in blue, while other Eukaryota are in black and Bacteria are brown. *Perkinsela* spp. group together and with diverse eukaryotes from the Alveolata, Stramenopiles, Rhizaria, and Opisthokonta, consistent with an ancestral origin of this protein. However, the bootstrap and posterior probabilities for the deeper nodes on the tree are generally weak. The scale bar shows the inferred number of amino acid substitutions per site.

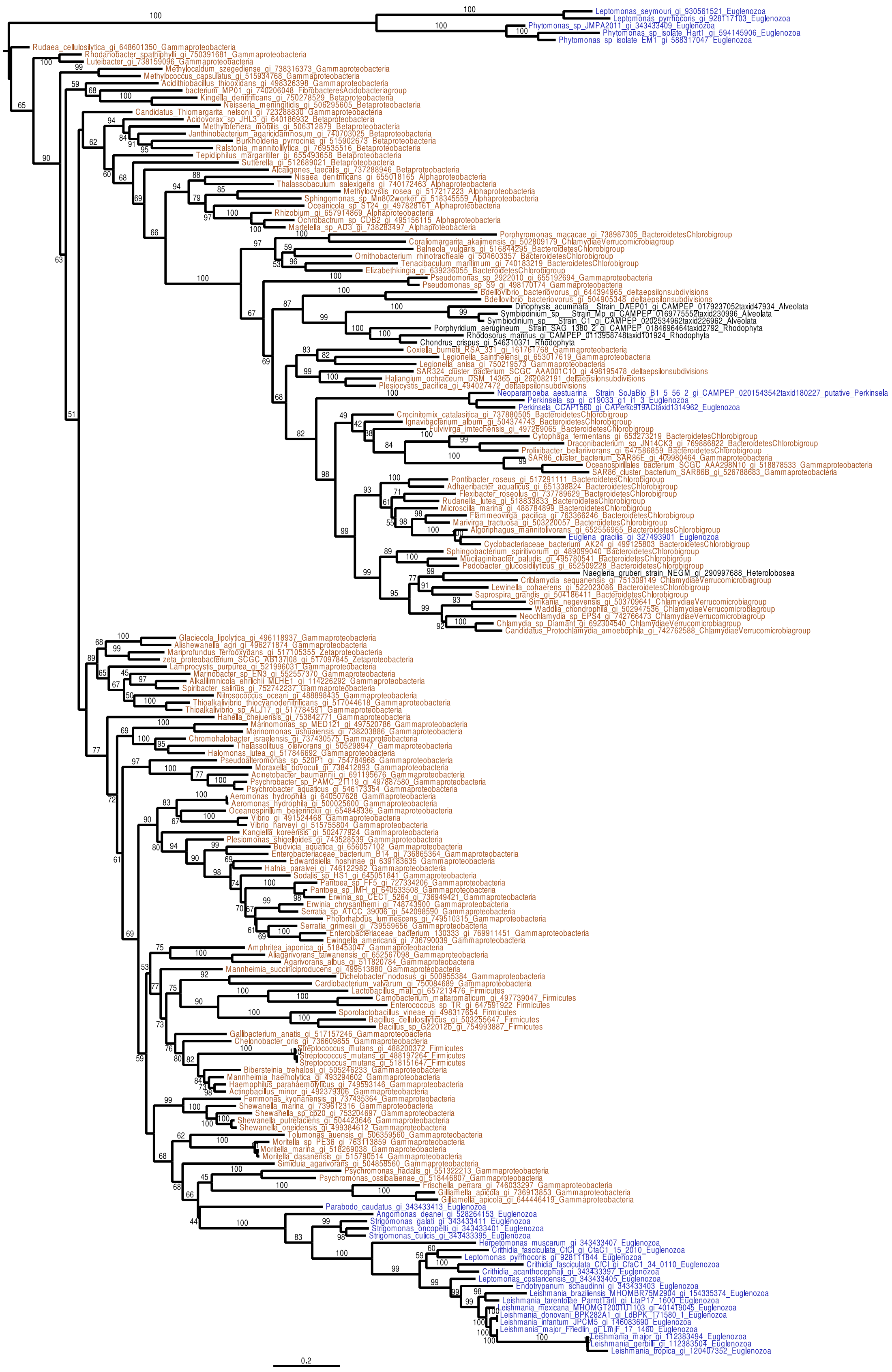

Figure S2.2: FeCH phylogenetic tree. The global tree shown was generated using IQTREE and the ultrafast bootstrap method with the LG4X model. The robustness of each branch is indicated with bootstrap values, with only those >50% displayed. Groups are colored according to their taxonomic

affiliation: Euglenozoa are in blue while other Eukaryotes are in black and Bacteria are brown. The scale bar shows the inferred number of amino acid substitutions per site. Three groups of euglenozoan sequences are found: (i) The *Phytomonas* spp. and *Leptomonas* spp. homologs, which are divergent in nature ; (ii) The *Perkinsela* spp. sequences, which branch in the same bacterial clade as those of *E. gracilis* and *N. gruberi* ; and (iii) a strongly supported monophyletic grouping of Strigomanidae and Leishmaniinae, weakly branching together with *Parabodo caudatus* (bootstrap value = 44%). The latter grouping is likely artefactual, based on the results of various other phylogenetic analyses (data not not shown; see also Figure S2.4).

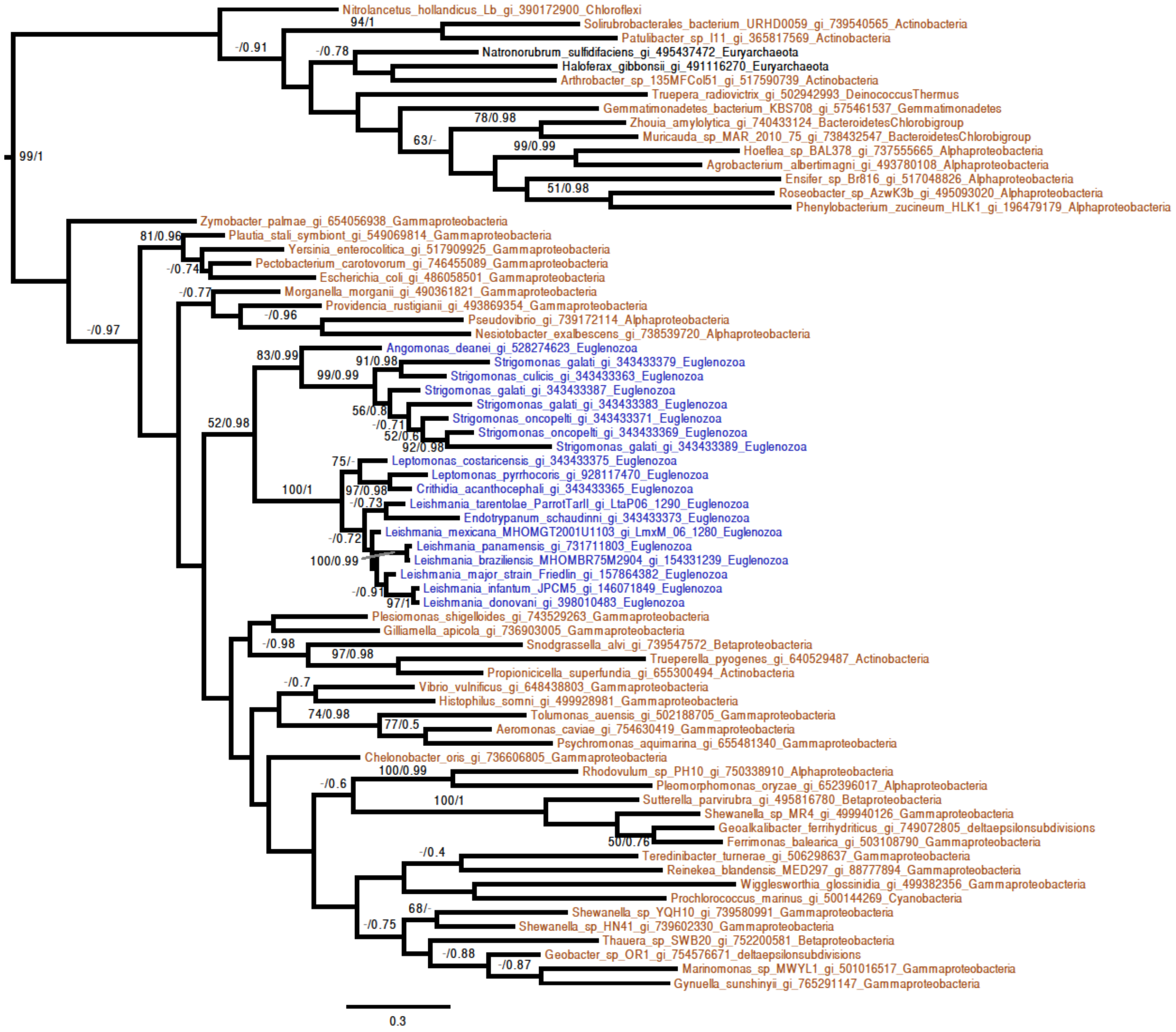

Figure S2.3: PPOX/HemG phylogenetic tree. The tree displayed is the consensus tree obtained with Phylobayes 4.1 with ML boostrap values (left) and bayesian posterior probabilities (left) mapped onto the nodes. Bootstrap values >50% are shown, while only posterior probabilities >0.6 are shown. Groups are colored according to taonomy: Euglenozoa are in blue while Bacteria and Archaea are brown and black, respectively. The Leishmaniinae and Strigomanidae sequences group together with high posterior probability (0.98). The Leishmaniinae and Strigomanidae appear to have acquired the HemG protein gene from the Gammaproteobacteria, perhaps in a common ancestor shared by these two kinetoplastid lineages. The scale bar shows the inferred number of amino acid substitutions per site.

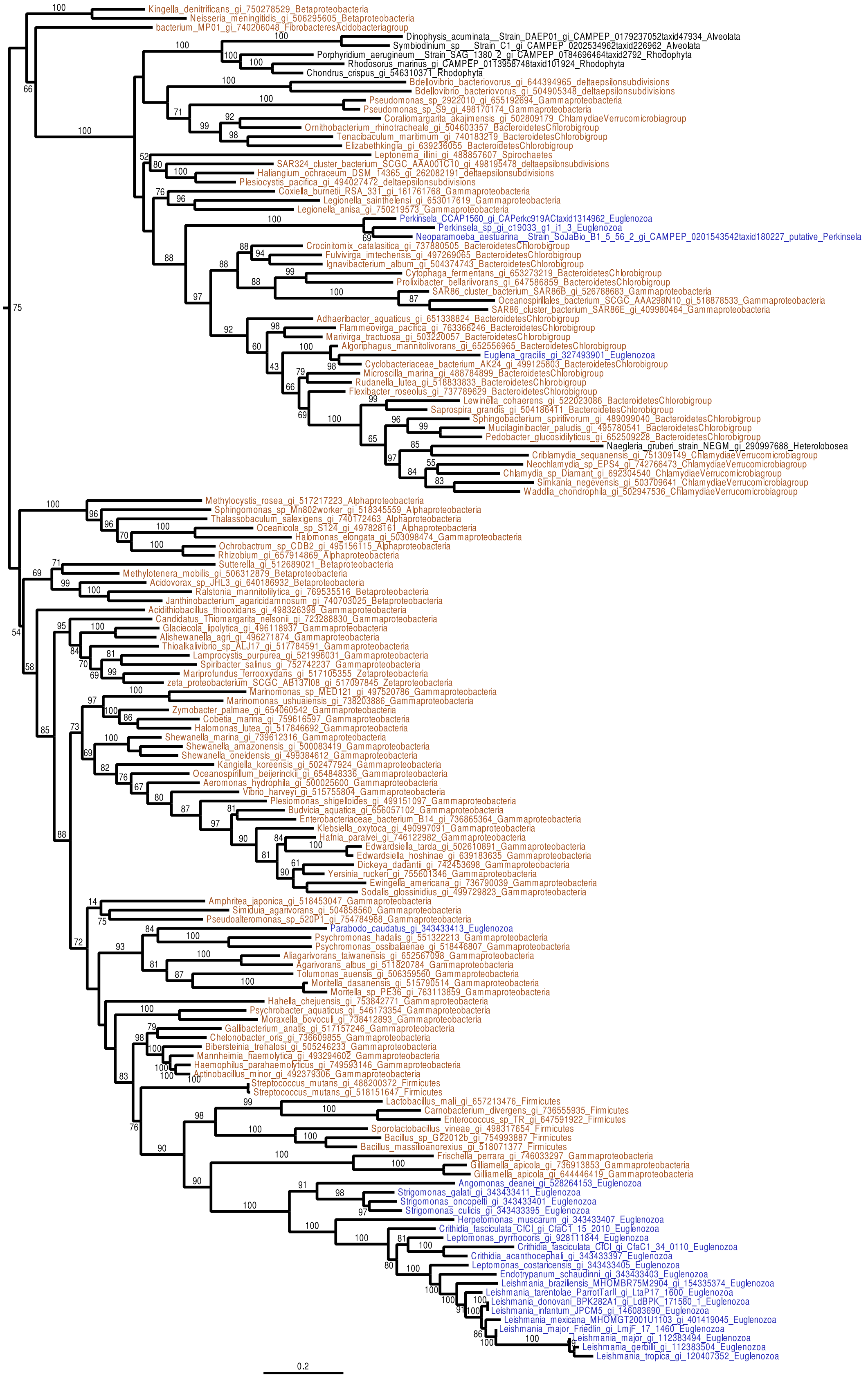

Figure S2.4: FeCH phylogenetic tree. The tree shown was generated with IQTREE and the ultrafast bootstrap method with the LG4X model. Bootstrap values >50% are shown. The tree was calculated from an alignment from which the divergent *Phytomonas* spp. and *Leptomonas* spp. homologs (see

Fig. S2.2) were removed. Groups are colored according to taxonomic affiliation: Euglenozoa are in blue, eukaryotes are in black and Bacteria are brown. The scale bar shows the inferred number of amino acid substitutions per site. In contrast to Figure S2.2, a grouping of Strigomanidae, Leishmaniinae, and *Parabodo caudatus* is not recovered. Instead, *Parabodo caudatus* branches robustly with a sub-group of Gammaproteobacteria (bootstrap value = 93%).

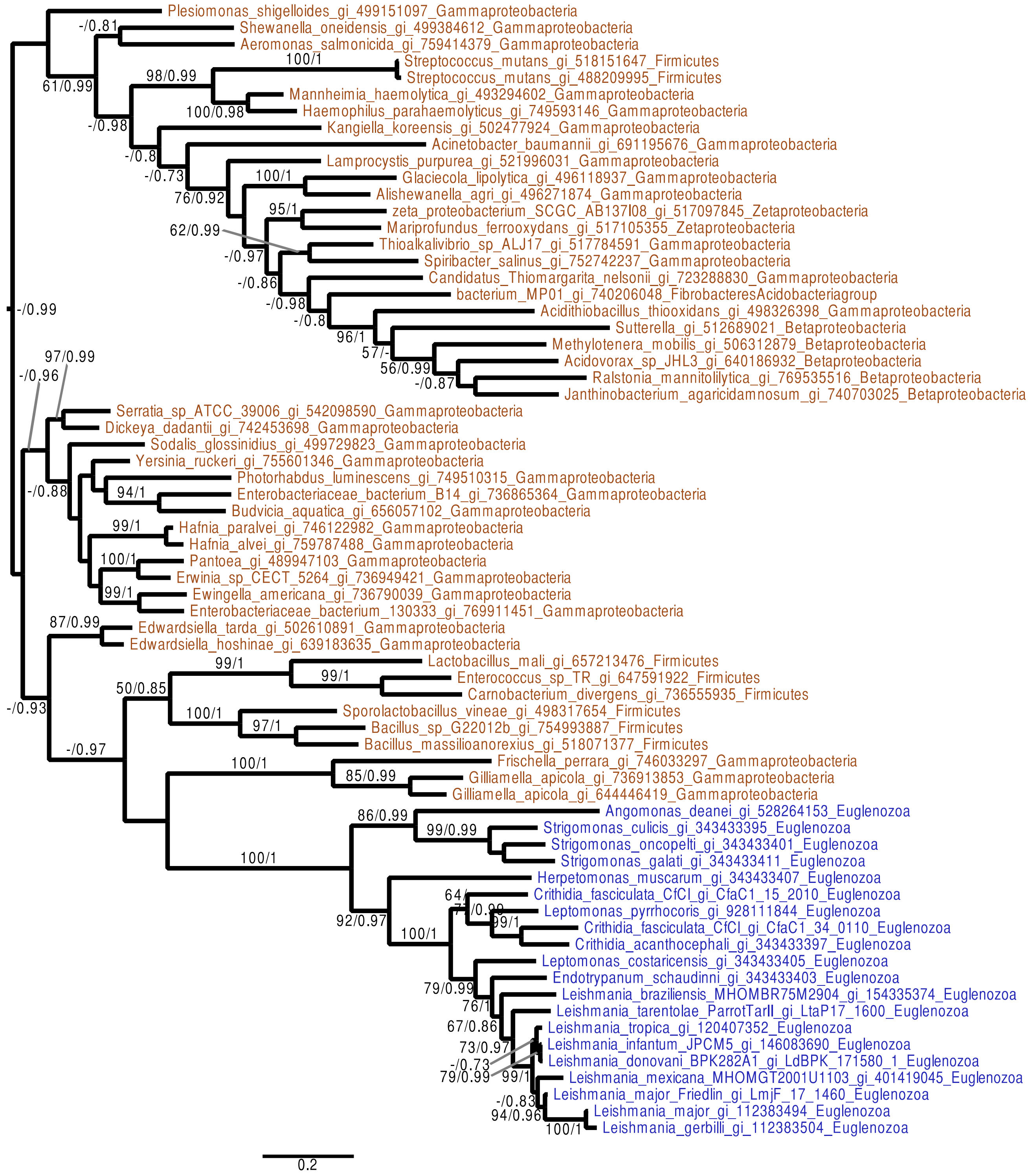

Figure S2.5: FeCH phylogenetic tree with taxon sampling focused on resolving the position of the Strigomanidae and Leishmaniinae. The tree shown is the consensus tree obtained with Phylobayes 4.1 with ML boostrap values (left) and bayesian posterior probabilities (right) mapped onto the nodes. Only bootstraps >50% are shown, while only posterior probabilities > 0.6 are shown. Groups are colored according to their taxonomic affiliation: Euglenozoa are in blue, Bacteria are brown. The Leishmaniinae and Strigomonadinae form a strongly supported monophyletic clade, most closely associated with Firmicutes and a subset of Gammaproteobacteria. The scale bar shows the inferred number of amino acid substitutions per site.

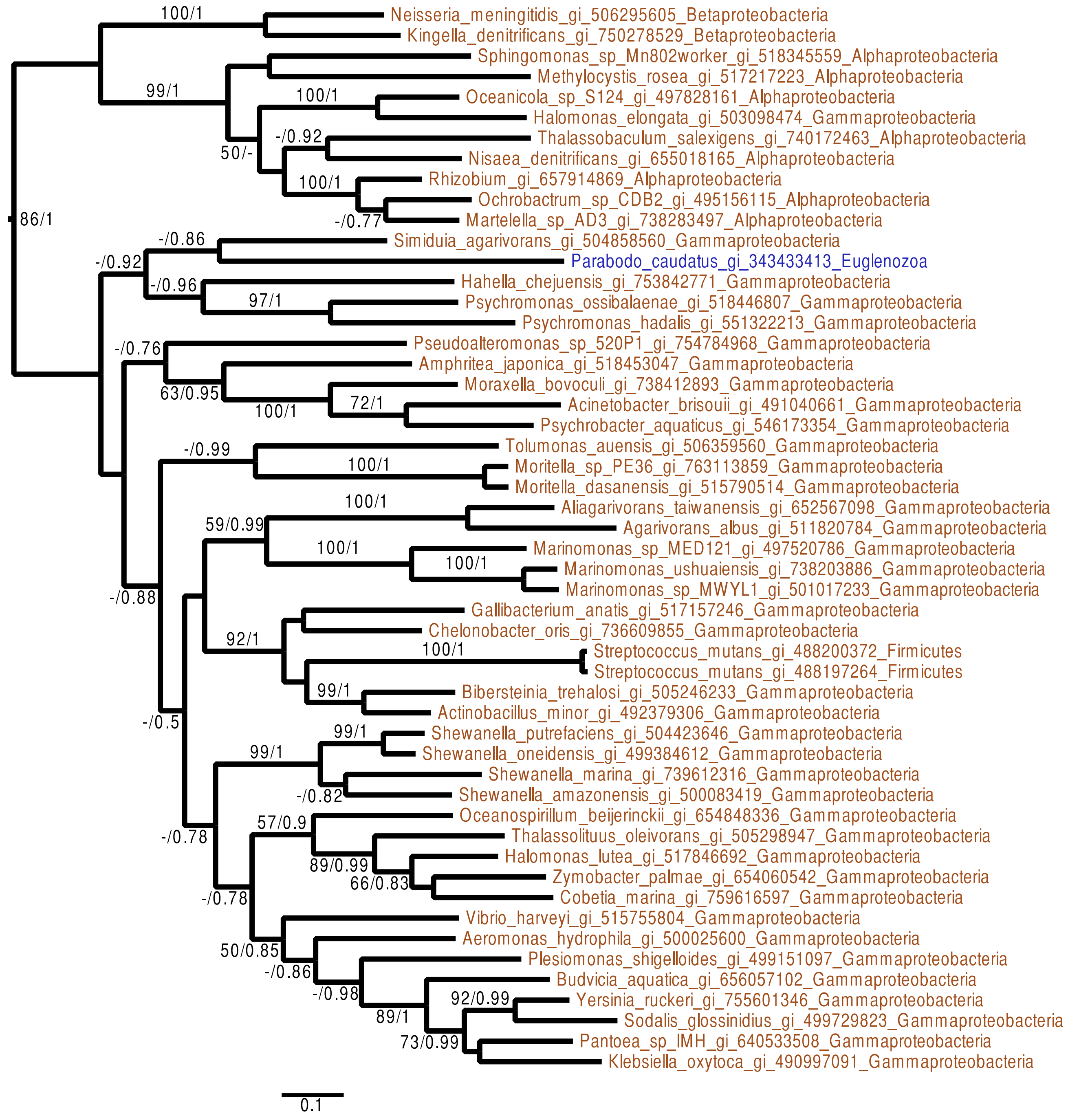

Figure S2.6: FeCH phylogenetic tree with taxonomic sampling focused on resolving the position of the *Parabodo caudatus* sequence. The tree shown is the consensus tree obtained with Phylobayes 4.1 with ML boostrap values (left) and bayesian posterior probabilities (right) mapped onto the nodes. Bootstrap values >50% are shown, while only posterior probabilities >0.6 are shown. The tree is rooted with the most distant group of bacteria. Groups are colored depending of their taxonomic affiliation: Euglenozoa are in blue and Bacteria are in brown. The *P. caudatus* FeCH branches amongst a large group of Gammaproteobacteria, suggestive of a LGT from Gammaproteobacteria to *P. caudatus*. The scale bar shows the inferred number of amino acid substitutions per site.

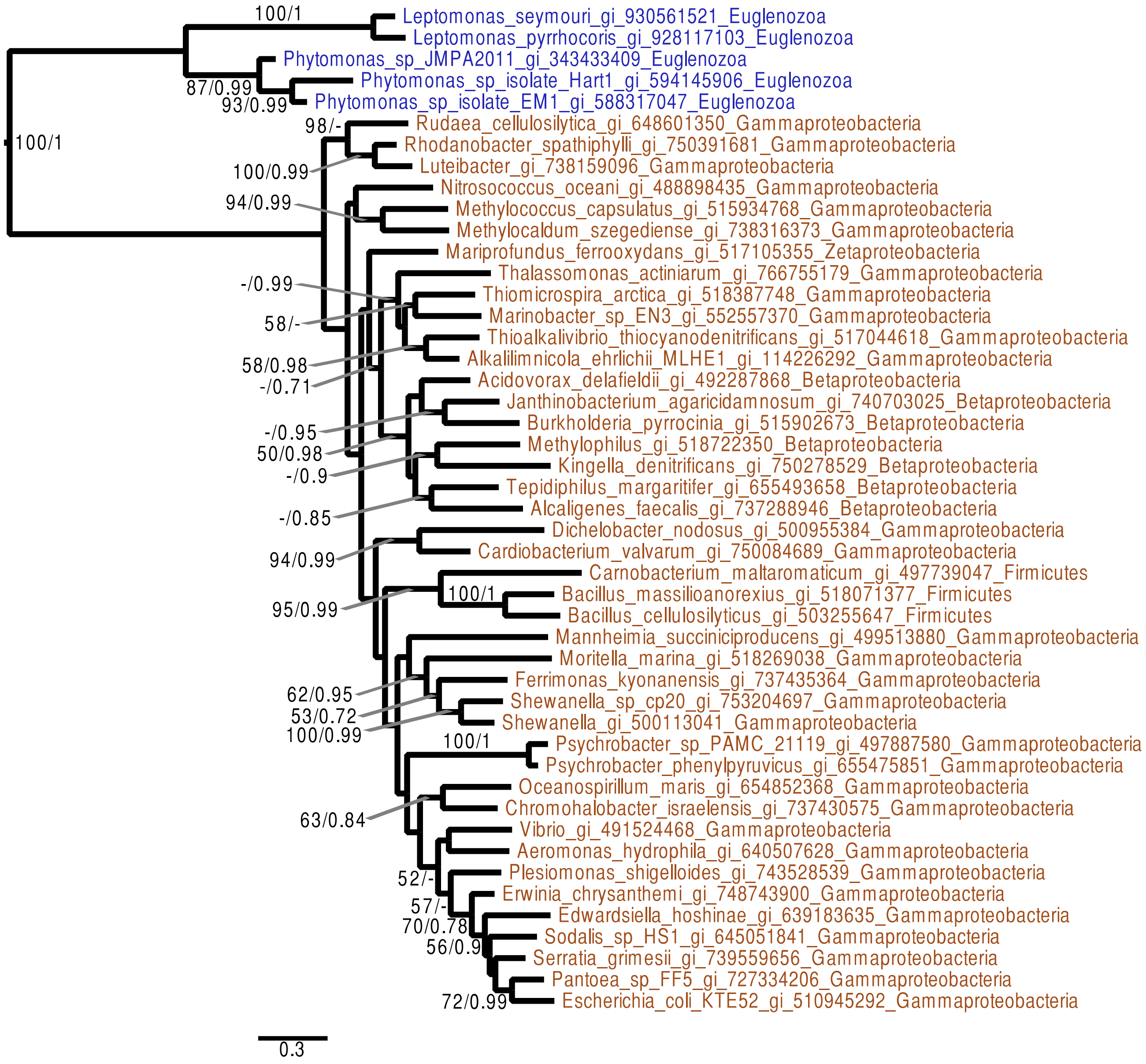

Figure S2.7: FeCH phylogenetic tree with taxonomic sampling focused on resolving the position of *Phytomonas* spp. and *Leptomonas* spp. The tree shown is the consensus tree obtained with Phylobayes 4.1 with ML boostrap values (left) and bayesian posterior probabilities (right) mapped onto the nodes. Bootstrap values >50% are shown, as are posterior probabilities >0.6. Euglenozoan sequences are in blue, Bacteria are brown. The *Phytomonas* spp. and *Leptomonas* spp. are highly divergent and difficult to assign to a specific bacterial group. The scale bar shows the inferred number of amino acid substitutions per site.

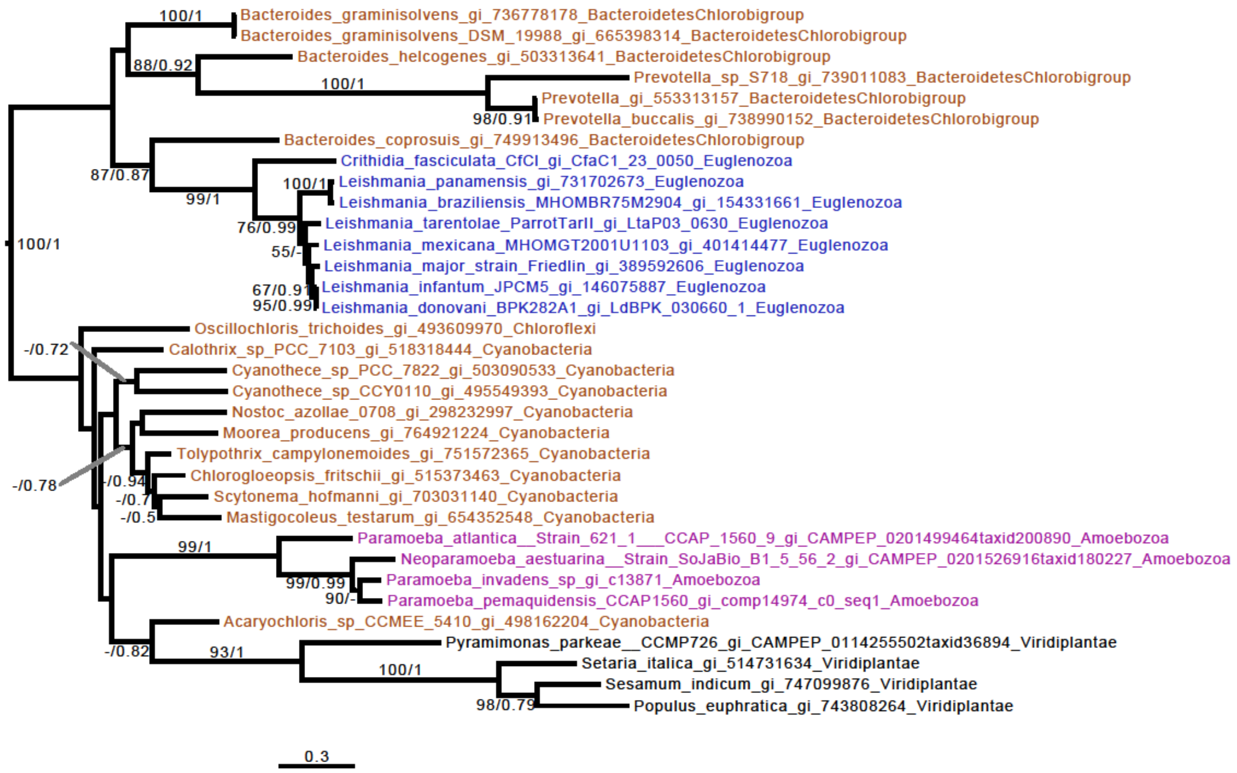

Figure S2.8: UROS phylogenetic tree. The tree shown is the consensus tree obtained with Phylobayes 4.1 with ML boostrap values (left) and bayesian posterior probabilities (right) mapped onto the nodes. Only bootstrap values >50% are shown, while only posterior probabilities >0.6 are shown. The tree is midpoint rooted. Groups are color coded according to their taxonomic affiliation: Euglenozoa are in blue, Bacteria are in brown, Amoebozoa are in purple, and other eukaryotic sequences are in black. After removal of the most distant bacterial sequences (Figure 8), we rebuilt the UROS phylogenetic tree in order to further test the lateral acquisition of the gene in Leishmaniinae from bacteria; the Leishmaniinae sequences branch robustly with the bacteroidetes sequences, and are distant from other eukaryotes found in the tree. The *Paramoeba* spp. UROS gene may have come from cyanobacteria by LGT. The scale bar shows the inferred number of amino acid substitutions per site.

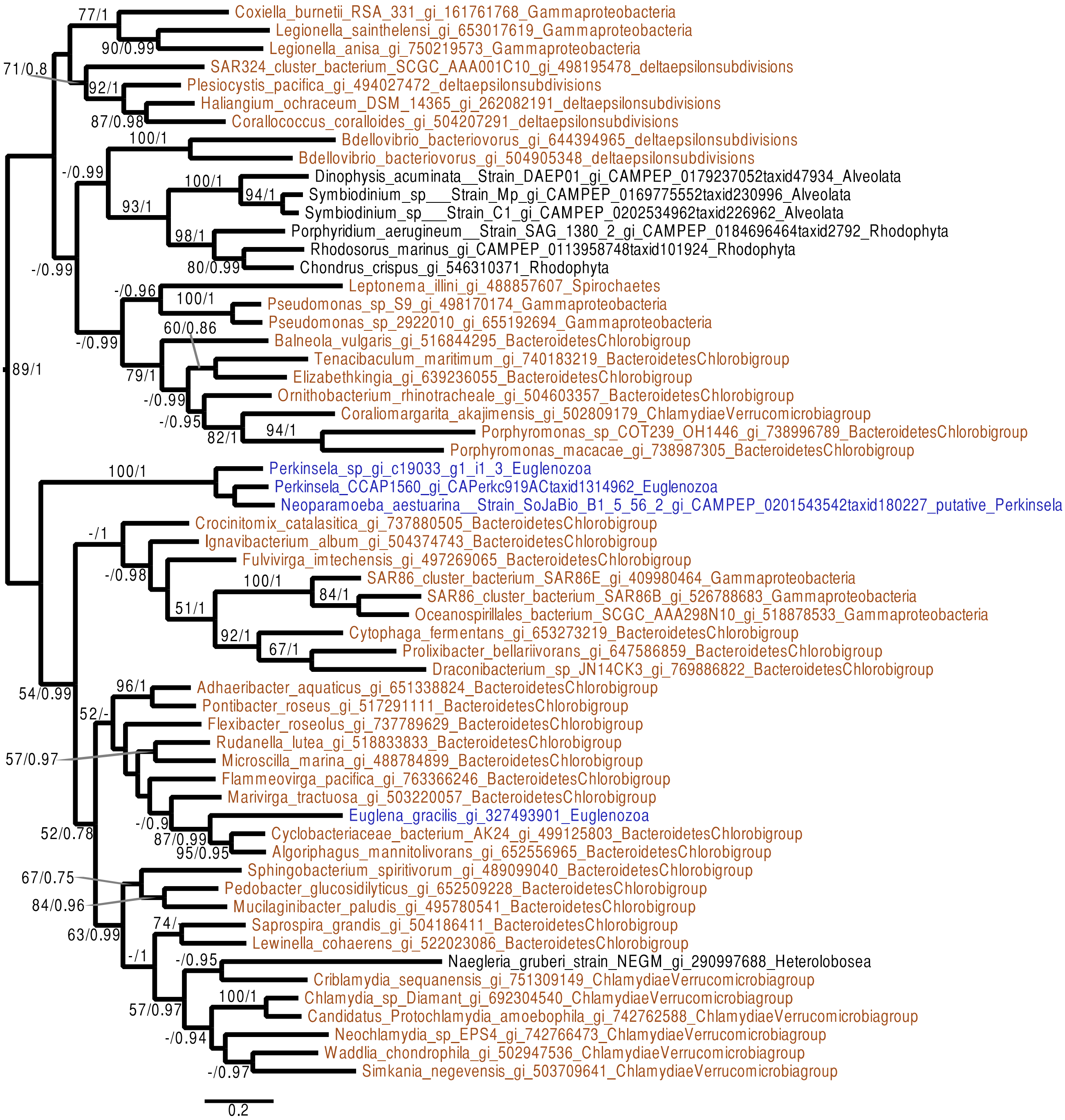

Figure S2.9: FeCH phylogenetic tree with taxonomic sampling focused on resolving the position of *Perkinsela* spp. sequences. The tree shown is the consensus tree obtained with Phylobayes 4.1 with ML boostrap values (left) and bayesian posterior probabilities (right) mapped onto the nodes. Bootstrap values >50% are shown, as are posterior probabilities >0.6. Groups are colored according to taxonomy: Euglenozoa are in blue and other Eukaryota are in black, while Bacteria are in brown. In this tree the *Perkinsela* spp. and *Euglena gracilis* FeCH sequences do not group together, and the *E. gracilis* homolog appears closely related to a sub-group of Bacteroidetes. In an attempt to better understand the relationship between *E. gracilis*, *N. gruberi* and *Perkinsela* spp., we removed the most distant sequences (Figure S2.10). The scale bar shows the inferred number of amino acid substitutions per site.

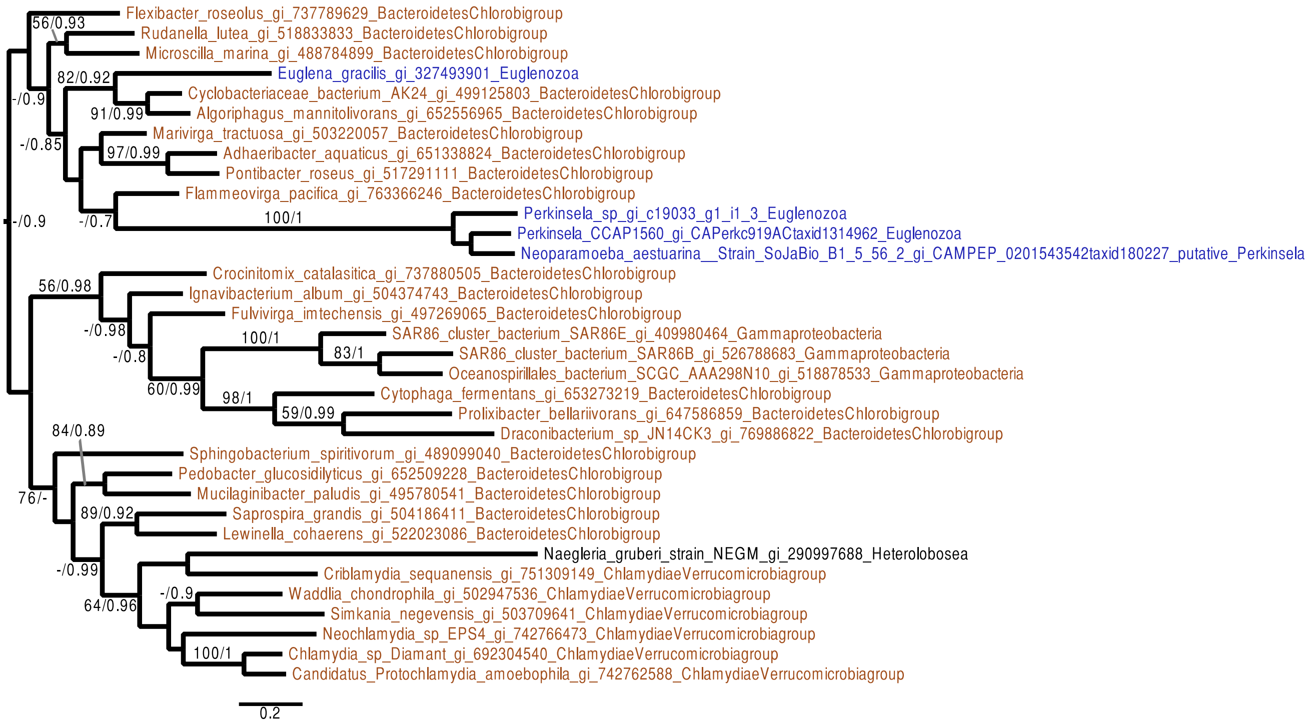

Figure S2.10: FeCH phylogenetic tree with taxonomic sampling focused on resolving the position of *Perkinsela* spp. sequences. The tree shown is the consensus tree obtained with Phylobayes 4.1 with ML boostrap values (left) and bayesian posterior probabilities (right) mapped onto the nodes. Only bootstrap values >50% are shown, while only posterior probabilities >0.6 are shown. The tree is rooted between *N. gruberi* and the Euglenozoa. Groups are colored depending of their taxonomic affiliation: Euglenozoa are in blue, other Eukaryota are in black, and Bacteria are shown in brown. The *Euglena gracilis* and Prokinetoplastina sequences branch with the same group of Bacteroidetes, while the *N. gruberi* sequence appears more distantly related. The scale bar shows the inferred number of amino acid substitutions per site.
